# The Herpes Simplex Virus Protein pUL31 Escorts Nucleocapsids to Sites of Nuclear Egress, a Process Coordinated by Its N-Terminal Domain

**DOI:** 10.1371/journal.ppat.1004957

**Published:** 2015-06-17

**Authors:** Christina Funk, Melanie Ott, Verena Raschbichler, Claus-Henning Nagel, Anne Binz, Beate Sodeik, Rudolf Bauerfeind, Susanne M. Bailer

**Affiliations:** 1 Institute for Interfacial Engineering and Plasma Technology (IGVP), University of Stuttgart, Stuttgart, Germany; 2 Max von Pettenkofer-Institut, Ludwig-Maximilians-University Munich, Munich, Germany; 3 Institute of Virology, Hannover Medical School, Hannover, Germany; 4 Institute of Cell Biology, Hannover Medical School, Hannover, Germany; 5 Fraunhofer Institute for Interfacial Engineering and Biotechnology (IGB), Stuttgart, Germany; University of Glasgow, UNITED KINGDOM

## Abstract

Progeny capsids of herpesviruses leave the nucleus by budding through the nuclear envelope. Two viral proteins, the membrane protein pUL34 and the nucleo-phosphoprotein pUL31 form the nuclear egress complex that is required for capsid egress out of the nucleus. All pUL31 orthologs are composed of a diverse N-terminal domain with 1 to 3 basic patches and a conserved C-terminal domain. To decipher the functions of the N-terminal domain, we have generated several Herpes simplex virus mutants and show here that the N-terminal domain of pUL31 is essential with basic patches being critical for viral propagation. pUL31 and pUL34 entered the nucleus independently of each other via separate routes and the N-terminal domain of pUL31 was required to prevent their premature interaction in the cytoplasm. Unexpectedly, a classical bipartite nuclear localization signal embedded in this domain was not required for nuclear import of pUL31. In the nucleus, pUL31 associated with the nuclear envelope and newly formed capsids. Viral mutants lacking the N-terminal domain or with its basic patches neutralized still associated with nucleocapsids but were unable to translocate them to the nuclear envelope. Replacing the authentic basic patches with a novel artificial one resulted in HSV1(17^+^)Lox-UL31-hbpmp1mp2, that was viable but delayed in nuclear egress and compromised in viral production. Thus, while the C-terminal domain of pUL31 is sufficient for the interaction with nucleocapsids, the N-terminal domain was essential for capsid translocation to sites of nuclear egress and a coordinated interaction with pUL34. Our data indicate an orchestrated sequence of events with pUL31 binding to nucleocapsids and escorting them to the inner nuclear envelope. We propose a common mechanism for herpesviral nuclear egress: pUL31 is required for intranuclear translocation of nucleocapsids and subsequent interaction with pUL34 thereby coupling capsid maturation with primary envelopment.

## Author Summary

Herpesviral capsid assembly is initiated in the host nucleus. Due to size constraints, newly formed nucleocapsids are unable to leave the nucleus through the nuclear pore complex. Instead herpesviruses apply an evolutionarily conserved mechanism for nuclear export of capsids called nuclear egress. This process is initiated by docking of capsids at the inner nuclear membrane, budding of enveloped capsids into the perinuclear space followed by de-envelopment and release of capsids to the cytoplasm where further maturation occurs. Two viral proteins conserved throughout the herpesvirus family, the membrane protein pUL34 and the phosphoprotein pUL31 form the nuclear egress complex that is critical for primary envelopment. We show here that pUL31 and pUL34 enter the nucleus independently of each other. pUL31 is targeted to the nucleoplasm where it binds to nucleocapsids via the conserved C-terminal domain, while its N-terminal domain is important for capsid translocation to the nuclear envelope and for a coordinated interaction with pUL34. Our data suggest a mechanism that is apparently conserved among all herpesviruses with pUL31 escorting nucleocapsids to the nuclear envelope in order to couple capsid maturation with primary envelopment.

## Introduction

Morphogenesis of herpesviral capsids is an intricate process initiated in the infected nucleus [1]. A fragile procapsid is formed and packaged with one copy of the viral genome that is generated by cleavage of replicated concatameric DNA molecules. During this process, the rather spherical procapsids change their conformation and mature into the icosahedral and more stable C capsids. These accumulate in large numbers in capsid assembly sites and in the nucleoplasm. Over time, the infected nuclei are enlarged, concurrently the capsids get dispersed, the host chromatin is marginalized, and the nuclear lamina is partially disintegrated [2–5]. How mature capsids are released from sites of assembly, and how they translocate from there to the nuclear envelope is not completely understood, and their mode of transport to the nuclear periphery is discussed controversially [5–9].

With a diameter of 125 nm, herpesviral nucleocapsids exceed the nuclear pore diameter forcing them to take a different route out of the nucleus. Nuclear egress involves primary envelopment of capsids at the inner nuclear membrane (INM) resulting in a transiently enveloped perinuclear particle followed by de-envelopment at the outer nuclear membrane (ONM) and release of capsids to the cytoplasm [10,11]. Nuclear egress of all herpesviruses is mediated by a group of conserved viral proteins. In Herpes simplex virus type 1 (HSV-1), pUL31, a nucleo-phosphoprotein [12], and pUL34, a type II membrane protein [13], are recruited to the INM where they form the nuclear egress complex (NEC; [13,14]). Both proteins are required for nuclear egress of capsids out of the nucleus since deletion of either NEC component leads to their nuclear retention concomitant with a defect in viral propagation [15,16]. Moreover, the NEC recruits several viral and cellular kinases to partially disintegrate the major host barriers, namely the chromatin and the nuclear lamina, and to provide access of capsids to the INM [17–21]. Current data on pUL34 and pUL31 interaction(s) support a temporally regulated and orchestrated sequence of events at the INM, e.g. docking of capsids at the nucleoplasmic face, initiation of membrane curvature, wrapping of capsids by the INM, completion of budding by membrane scission and release of enveloped capsids into the perinuclear space [22–27]. *In vivo*, co-expression of the two NEC proteins in absence of any other viral protein is sufficient to form and accumulate empty vesicles in the perinuclear space [28,29]. Recently, insights into the membrane-associated NEC activity have been obtained by *in vitro* systems [30,31]. Recombinant HSV-1 pUL31 and pUL34 form ordered coats on artificial membranes and can induce membrane curving, invaginations, and vesicle formation. Thus, the NEC represents the minimal virus-encoded membrane-budding machinery with an intrinsic activity to drive membrane budding and scission of vesicles [30,31]. During infection, the situation is more complex due to the presence of other viral and cellular factors and their spatio-temporal regulation. Among them are the nonessential HSV-1 protein kinase pUS3 [32], the viral proteins pUL47 [33] and ICP22/pUS1 [34] as well as numerous host factors [33]. In addition to its well documented role in primary envelopment of nucleocapsids [18,25,26,35], pUL31 may assist in viral genome cleavage/packaging [15,36–41] and thus link capsid maturation to nuclear egress. Several studies have reported a preferred nuclear egress of C capsids over A or B capsids ([10,11]; and references therein); however, the molecular mechanism of these sorting events is poorly understood. The minor capsid proteins pUL25 and pUL17 that physically interact with pUL31 [38,39,41,42] are candidates to contribute to this quality control of nuclear capsid egress [10,11,27,43,44].

**Fig 1:**
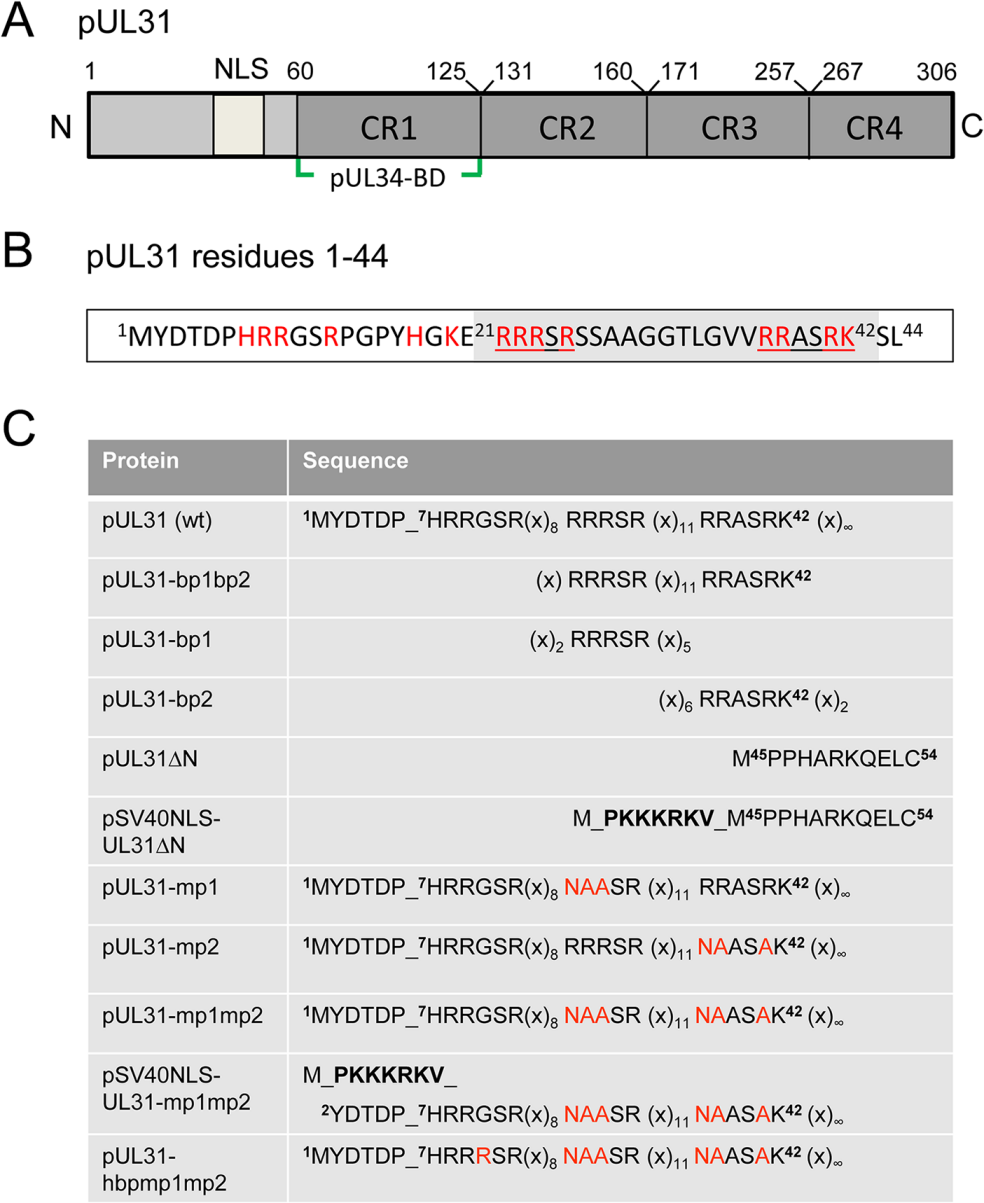
Domain organization and N-terminal mutants of pUL31. (A) Graphic depiction of HSV-1 pUL31 composed of the N-terminal variable domain carrying a putative nuclear localization signal (NLS) and the C-terminal domain with conserved regions CR1, CR2, CR3, CR4 and a pUL34-binding site (pUL34-BD). (B) Sequence of the first 44 amino acids of HSV-1 pUL31. Basic amino acids are highlighted in red, the bipartite NLS (residues 21–42) is shaded in grey and its basic patches bp1 and bp2 are underlined. (C) Mutants of the N-terminal domain of pUL31 generated in this study. Exchanged residues are shown in red, the SV40NLS is indicated in bold letters, (x) indicate numbers of interspersed residues. Mutated basic patches (mp) and the hyperbasic patches (hbp) can be deduced.

Orthologous pUL31 proteins share several features. The larger C-terminal domain can be divided into four conserved regions CR1 to 4 [45,46] with CR1 of all pUL31 orthologs containing a binding site for the respective pUL34 ortholog (Fig 1A; [45–47], and references therein); however, additional binding sites are likely to exist in their C-terminal domain [25,26,48–50]. In contrast, the smaller N-terminal domains are variable and enriched in basic residues clustered in several patches (red in Fig 1B). Furthermore, a putative classical bipartite nuclear localization signal (NLS; [45,50–52]) has been identified by *in silico* analysis (Fig 1A, grey in Fig 1B). To characterize the functions of the N-terminal domain of pUL31 reported to be phosphorylated by the US3 protein kinase [18], we generated a series of HSV-1 mutants with a particular focus on the basic patches (Fig 1C). We identified a classical bipartite NLS embedded in the N-terminal domain that was however not required for nuclear import of pUL31 during HSV-1 infection. Furthermore, we show here that pUL31 and pUL34 entered the nucleus independently of each other via separate routes. pUL31 lacking the N-terminal domain associated with capsids in the nucleoplasm but was unable to support nuclear egress and viral replication. A considerable amount of pUL31ΔN was retained in the cytoplasm if co-expressed with pUL34 suggesting that these proteins had prematurely interacted, and that the N-terminal domain of pUL31 controls the interaction with pUL34. Interestingly, while the C-terminal domain of pUL31 was sufficient to interact with nucleocapsids, the N-terminal domain was required for translocation of capsids from the nucleoplasm to the nuclear envelope and for viral propagation. Together, our data suggest a highly regulated sequence of events during nuclear egress: pUL31 is initially targeted to nuclear sites of capsid assembly and then escorts the nucleocapsids to the nuclear envelope for primary envelopment, a process coordinated by the N-terminal domain of pUL31.

## Materials and Methods

### Cells, viruses, and general methods

Hep2 (ATCC-No. CCL-23), HeLa (ATCC-No. CCL-2) and Vero cells (ATCC-No. CCL-81) were cultured as described previously [53]. HSV1(17^+^)Lox was used for all experiments [54,55]. The HSV-1 strain 17^+^ (kindly provided by D. J. McGeoch) and pHSV1(17^+^)Lox [54–56] were used for PCR amplification. HSV-1 propagation, titration and kinetics were done as described previously [13,53]. Plasmid transfection was performed using Effectene Transfection Reagent (Qiagen), while BAC transfection was done using Lipofectamine 2000 (Invitrogen). The yeast 2-hybrid method (Y2H; [53,57]), the NEX-TRAP assay [58] and the LUMIER assay [59,60] were described previously.

### Plasmids

Cloning was performed by classical restriction or Gateway recombination according to the manufacturer’s protocol (Gateway, Invitrogen). Single base pair exchanges were introduced using the QuikChange Site-directed Mutagenesis Kit (Stratagene) and verified by sequencing. Constructs encoding pUL31 or mutants thereof were cloned into pCR3-N-myc destination vectors. Constructs encoding maltose-binding protein (MBP)-UL34 were cloned into the pCR3-MBP destination vector similar to the plasmid encoding Strep-pUL34 described previously [13]. The plasmids encoding bp1 (basic patch 1), bp2 or bp1bp2 were cloned using the plasmid EYFP (Clontech). Primers used to generate plasmids encoding EYFP-UL31-bp1bp2,-bp1,-bp2, and pUL31-mp1 (mutant patch 1),-mp2,-mp1mp2,-hbpmp1mp2, pSV40NLS-UL31-mp1mp2, pUL31ΔN, and pSV40NLS-UL31ΔN (Table 1) are described in Table 2. The plasmid EYFP-Nuc (Clontech) was used as control. Plasmids used for the yeast 2-hybrid (Y2H) and the LUMIER assays [59] and the plasmid EYFP-FRB-pUL31 [58] have been described before.

**Table 1:** N-terminal mutants of pUL31.

Protein	Protein sequence	BAC pHSV1(17^+^)	Growth
pUL31	^7^HRRGSR(x)_8_RRRSR(x)_11_RRASRKSL^44^	Lox-UL31	+++
pUL31ΔN	M_^45^PPHARKQELC^55^	Lox-UL31ΔN	-
pSV40NLS-UL31ΔN	M_**PKKKRKV**_M^45^PPHARKQELC^55^	Lox-SV40NLS-UL31ΔN	-
pUL31-mp1	^7^HRRGSR(x)_8_ NAASR (x)_11_RRASRKSL^44^	Lox-UL31-mp1	++
pUL31-mp2	^7^HRRGSR(x)_8_ RRRSR (x)_11_NAASAKSL^44^	Lox-UL31-mp2	++
pUL31-mp1mp2	^7^HRRGSR(x)_8_NAASR (x)_11_NAASAKSL^44^	Lox-UL31-mp1mp2	-
pUL31	^7^HRRGSR(x)_8_RRRSR (x)_11_RRASRKSL^44^	Lox-UL31-mp1mp2 rescue	+++
pUL31	^7^HRRGSR(x)_8_RRRSR (x)_11_RRASRKSL^44^	Lox-UL31-mp1mp2 rev	+++
pSV40NLS-UL31-mp1mp2	M_**PKKKRKV_** ^2^YDTDPHRRGSR(x)_8_NAASR (x)_11_NAASAKSL^44^	Lox-SV40NLS-UL31-mp1mp2	-
pSV40NLS-UL31	M_**PKKKRKV**_^2^YDTDPHRRGSR(x)_8_RRRSR (x)_11_RRASRKSL^44^	Lox-SV40NLS-UL31-mp1mp2 rev	+++
pUL31-hbp1mp1mp2	^7^HRRRSR(x)_8_ NAASR (x)_11_NAASAKSL^44^	Lox-UL31-hbp1mp1mp2	+

**Table 2:** Oligonucleotides used for general cloning and site-directed mutagenesis.

Nr.	Name	Sequence (5‘→3‘)
1	EYFP-UL31-bp1bp2 for	TCAGATCCGCTAGCGCTACCGGTCGCCACCATGGTGAGCAAGGGCGAG
2	EYFP-UL31-bp1bp2 rev	TTTGGATCCCTACTTCCGGGAGGCCCGACGCACCACGCCCAGAGTCCCGCCGGCCGC
3	EYFP-UL31-bp1 for	ATCCGCTAGCGCTACCGGTCGCCACCATGGTGAGC
4	EYFP-UL31-bp1 rev	TTTGGATCCCTAGCCGGCCGCAGAGGAGCGCGACCGCCGGCGCTCCTTAGCACGAGATCTG
5	EYFP-UL31-bp2 for	ATCCGCTAGCGCTACCGGTCGCCACCATGGTGAGC
6	EYFP-UL31-bp2 rev	TTTGGATCCCTACAGGCTCTTCCGGGAGGCCCGACGCACCACGCCCAGAGTCCCAGCACGAGATCTG
7	UL31 for	AAAAAGCAGGCTCCGCCATGTATGACACCGACCCCCATC
8	UL31 rev	AGAAAGCTGGGTCCTACGGCGGAGGAAACTC
9	UL31-mp1 for	CCCTATCACGGCAAGGAGAACGCGGCGTCGCGCTCCTCTGCG
10	UL31-mp1 rev	CGCAGAGGAGCGCGACGCCGCGTTCTCCTTGCCCTGATAGGG
11	UL31-mp2 for	CTCTGGGCGTGGTGAATGCGGCCTCCGCGAAGAGCCTGCCGCC
12	UL31-mp2 rev	GGCGGCAGGCTCTTCGCGGAGGCCGCATTCACCACGCCCAGAG
13	SV40NLS-UL31-mp1mp2 for	AAAAAGCAGGCTCCGCCATGCCAAAAAAGAAGAGAAAGGTATATGACACCGACCCCCATC
14	SV40NLS-UL31-mp1mp2 rev	AGAAAGCTGGGTCCTACGGCGGAGGAAACTC
15	UL31ΔN for	AAAAAGCAGGCTCCGCC**ATG**CCGCCTCACGCCCGCAAAC
16	UL31ΔN rev	AGAAAGCTGGGTCCTACGGCGGAGGAAACTC
17	SV40NLS-UL31ΔN for	AAAAAGCAGGCTCCGCCATGCCAAAAAAGAAGAGAAAGGTACCGCCTCACGCCCGCAAAC
18	SV40NLS-UL31ΔN rev	AGAAAGCTGGGTCCTACGGCGGAGGAAACTC
19	UL31-hbpmp1mp2 for	GACCCCCATCGCCGCCGCTCCCGGTCCGGGCCCTATCACG
20	UL31-hbpmp1mp2 rev	CGTGATAGGGCCCGGACCGGGAGCGGCGGCGATGGGGGTC

Nucleotides altered by site directed mutagenesis are underlined.

### BAC mutagenesis

The HSV-1 UL31 mutants were generated using pHSV1(17^+^)Lox [54–56] and a modified *galK* positive counterselection scheme essentially as described ([13] and Striebinger *et al*., in revision). First, the non-overlapping coding region of UL31 (Nucleotides 9 to 865) was replaced by a *galK-kan* cassette, which had been amplified using the pGPS-*galK-kan* plasmid and primers equipped with 50bp homologies flanking the UL31 locus (Table 3: H5-UL31/*galk* and H3-UL31/*galk*). In a second step, the *galK-kan* cassette was substituted with a UL31 region encoding either wild type (wt) pUL31, pUL31ΔN, pSV40NLS-UL31ΔN, pUL31-mp1 (mutant patch 1), pUL31-mp2, pUL31-mp1mp2, or pUL31-hyperbasic patch 1 (hbp1)mp1mp2 (Fig 1C; Tables 1, 3 and 4). To rescue the ΔUL31/*galk* intermediate, the *galK-kan* cassette was replaced by the wt UL31 sequence. To reverse the pHSV1(17^+^)Lox-UL31-mp1mp2 to a pUL31 wt sequence, a two-step recombination process was applied resulting in pHSV1(17^+^)Lox-UL31-mp1mp2 revertant (*rev*). For PCR amplification, mutant plasmids that had been generated by site-directed mutagenesis using specific primers (Table 2) were used as templates. Details of BAC mutants are presented in Tables 1 and 4. Direct insertion of the SV40NLS coding sequence at the 5´end of UL31 would have perturbed the 3´coding sequence of UL32. To leave the UL32 coding sequence intact, a BAC was generated in which *galK-kan* was inserted into the UL31 locus while the original start site of UL31 was inactivated without changing the amino acid sequence of pUL32. Upon insertion of the coding sequence of pSV40NLS-UL31-mp1mp2, the overlapping 8 bp of UL31 and UL32 were duplicated. To reverse the pHSV1(17^+^)Lox-SV40NLS-UL31-mp1mp2 to a UL31 wt sequence, a two-step recombination process was applied resulting in pHSV1(17^+^)Lox-SV40NLS-UL31-mp1mp2 revertant (*rev*). This revertant still carries the 8 bp duplication of the 5´ UL31 region as well the mutated original start codon of UL31 (Tables 3–4). All BAC sequences were validated by sequencing of the DNA regions targeted by mutagenesis and by restriction pattern analysis of the entire BAC backbone. The pHSV1(17^+^)Lox strains were reconstituted by transfecting BAC DNA into Vero cells using Lipofectamine 2000 according to the manufacturer´s instructions (Invitrogen).

**Table 3:** Oligonucleotides used for BAC-mutagenesis.

Nr.	Oligo	Sequence (5‘→3‘)
1	H5-UL31/*galk*	CGGAGGAAACTCGTCGAATGTTGCATAGAGCCTTTGATACTCTAGCATGACCTGTTGACAATTAATCATCGGCA
2	H3-UL31/*galk*	CTCGATCTCGCTCCTGTCCCTGGAGCACACCCTGTGTACCTATGTATGAGCCAGTGTTACAACCAATTAACC
3	H3-UL31/*galk rev*	CTCGATCTCGCTCCTGTCCCTGGAGCACACCCTGTGTACCTACGTATGAGCCAGTGTTACAACCAATTAACC
4	H5-UL31 wt and mt	CTACGGCGGAGGAAACTCGTCGAATGTTGCATAGAGCCTTTGATACTCTAGCATG
5	H3-UL31 wt and mt	CTTAGACGCCACTCGATCTCGCTCCTGTCCCTGGAGCACACCCTGTGTACCT**ATG**TATGACACAGACCCCCATC
6	H3-SV40NLS-UL31-mp1mp2	GACGCCACTCGATCTCGCTCCTGTCCCTGGAGCACACCCTGTGTACCTa*c*gtatga**ATG** **CCAAAAAAGAAGAGAAAG**
7	H3-SV40NLS-UL31-mp1mp2*rev*	CTCGATCTCGCTCCTGTCCCTGGAGCACACCCTGTGTACCTa*c*gtatga**ATG**TATGACACCGACCCC
8	H3-UL31-hbpmp1mp2 *rev*	CCTGGAGCACACCCTGTGTACCT**ATG**TATGACACCGACCCCCATCGCCGCCGCTCCCGGTCCGGGCCCTATCACGGCAAGG
9	H3-UL31ΔN _45–306_	GACGCCACTCGATCTCGCTCCTGTCCCTGGAGCACACCCTGTGTACCTacgtatga**ATG**CCGCCTCACG
10	H3-SV40NLS-UL31ΔN _45–306_	GATCTCGCTCCTGTCCCTGGAGCACACCCTGTGTACCT**ATG**TATGA**ATG** **CCAAAAAAGAAGAGAAAGGT**
11	BAC-UL31-Sequencing	GACGTATCAGGTATTTGTACCAAAGCC

Homology to the *galK-kan* selection cassette is underlined.

Coding sequence of the SV40NLS is bold, Start sites are bold and underlined.

Duplications are indicated by small letters.

**Table 4:** BAC mutagenesis.

Nr.	BAC	Inserted Fragment / Primer / Template	Recipient BAC
1	pHSV1(17^+^)Lox	*-*	
2	pHSV1(17^+^)Lox- ΔUL31/*galk*	*galK-kan* in UL31 locus (bp 9–865) (PCR: Oligos Nr.1/2; Table 3, *Template* pGPS-*galK-kan*)	1
3	pHSV1(17^+^)Lox—UL31wt (*rescue*)	UL31 wt (PCR: Oligos Nr.4/5; Table 3; *Template* pHSV-1(17^+^)Lox)	2
4	pHSV1(17^+^)Lox—UL31ΔN	UL31ΔN (PCR: Oligos 4/9; Table 3; *Template* Lox)	2
5	pHSV1(17^+^)Lox—SV40NLS-UL31ΔN	SV40NLS-UL31ΔN (PCR: Oligos 4/10; Table 3; *Template Lox*	2
6	pHSV1(17^+^)Lox—UL31-mp1mp2	UL31-mp1mp2 (PCR: Oligos Nr.4/5; Table 3; *Template* pDONR207-UL31-mp1mp2)	2
7	pHSV1(17^+^)Lox—SV40NLS-UL31-mp1mp2	SV40NLS-UL31-mp1mp2 (PCR: Oligos Nr.4/6; Table 3; *Template* pCR3-N-*c-myc*-SV40NLS-UL31-mp1mp2)	2
8	pHSV1(17^+^)Lox- ΔUL31-mp1mp2/*galk*	*galK-kan* in UL31-mp1mp2 locus (PCR: Oligos Nr.1/2; Table 3; *Template* pGPS-*galK-kan*)	6
9	pHSV1(17^+^)Lox—UL31-mp1mp2 *rev*	UL31 wt (PCR: Oligos Nr.4/5; Table 3; *Template* pHSV-1(17^+^)Lox)	8
10	pHSV1(17^+^)Lox- ΔSV40NLS-UL31-mp1mp2/*galk*	*galK-kan* in SV40NLS-UL31-mp1mp2 locus (PCR: Oligos Nr.1/3; Table 3; *Template* pGPS-*galK-kan*)	7
11	pHSV1(17^+^)Lox- SV40NLS-UL31-mp1mp2 *rev*	UL31 wt (PCR: Oligos Nr.4/7; Table 3; *Template* pHSV-1(17^+^)Lox)	10
12	pHSV1(17^+^)Lox—UL31-mp1	UL31-mp1 (PCR: Oligos Nr.4/5; Table 3; *Template* pDONR207-UL31-mp1)	2
13	pHSV1(17^+^)Lox—UL31-mp2	UL31-mp2 (PCR: Oligos Nr.4/5; Table 3; *Template* pDONR207-UL31-mp2)	2
14	pHSV1(17^+^)Lox—UL31-hbpmp1mp2	UL31-hbpmp1mp2 (PCR: Oligos Nr. Nr.4/8; Table 3; *Template* pDONR207-UL31-hbpmp1mp2)	2

### Immunofluorescence microscopy

Hep2, HeLa or Vero cells grown on coverslips, either transfected or infected, were fixed with 2% formaldehyde/PBS (15 min, room temperature) and permeabilized with 0.5% Triton X-100 (5 min, 4°C). Binding of antibodies to the HSV-1 Fc-receptor like proteins gE/gI was blocked with human blood sera of HSV-1 negative individuals/PBS for at least 3 h at room temperature [13]. Mouse monoclonal antibodies anti-myc (clone 9E10; kindly provided by J. von Einem), anti-MBP (NEB), anti-ICP0 (Santa Cruz), anti-ICP8 (kindly provided by R. Heilbronn) and anti-VP5 (clone 8F5; kindly provided by J. Brown) as well as rabbit anti-pUL31 and anti-pUL32 antibodies (kindly provided by B. Roizman and J. Baines [49]), anti-gM antibodies (kindly provided by T. Mettenleiter), and anti-pUL34 antibodies [13,53] were used. Anti-mouse and anti-rabbit fluorescently labelled secondary antibodies were from Invitrogen. Cells were examined using a confocal laser scanning microscope (LSM710; Zeiss, Oberkochen, Germany, or TCS SP5; Leica, Mannheim, Germany). Pictures were processed using Adobe Photoshop (Adobe) and Zen-Lite (Zeiss, Oberkochen, Germany). Fluorescence was measured along a 1 pixel thick and 6 μm long line using the "plot profile" tool of the software ImageJ (version 1.48K) on 8 bit images (Zeiss LSM710) taken with a 63x objective, NA 1.4, a pinhole aperture of 1 Airy unit, and a pixel size of 78 x 78 nm.

### Amylose-affinity purification

To evaluate complex formation between pUL31 and pUL34, 3.5 x 10^6^ HeLa cells were transfected with single plasmids encoding MBP-pUL34, myc-pUL31 or myc-pUL31ΔN, or a combination of a plasmid encoding MBP-pUL34 with one either encoding myc-pUL31 or myc-pUL31ΔN using Effectene according to the manufacturer´s protocol (Qiagen). Twenty-four hours post transfection (hpt), the cells were washed with ice-cold PBS, incubated for 20 min with ice-cold lysis buffer (20 mM Tris-HCl pH8, 150 mM NaCl, 10% (v/v) glycerol, 0.5% (v/v) Triton X-100, 2 mM EDTA, with complete Protease-Inhibitor Cocktail (Roche)). The lysates were pre-cleared by centrifugation (4°C, 12 000 rpm, 10 min) and incubation with Protein A Sepharose beads (GE Healthcare) for 10 min at 4°C. Following centrifugation (4°C, 5300 rpm, 10 min), the lysates were incubated with prewashed Amylose Resin (NEB). After incubation for 1 hour at 4°C on a rotating wheel, the supernatant was removed, and the beads were washed 3x using ice-cold lysis buffer. Proteins were released from the resin by incubation with 4x Lämmli buffer (room temperature, 15 min) and analyzed by SDS-PAGE followed by Western blotting using anti-MBP antibodies and anti-myc antibodies and peroxidase-conjugated secondary antibodies.

### Electron microscopy

Vero cells were seeded onto coverslips 1 day prior to infection. The cells were pre-cooled for 20 min on ice, and incubated with HSV-1 at 1 pfu/cell in CO_2_-independent medium containing 0.1% (w/v) BSA for 2 h on ice on a rocking platform as described previously [54,61]. The cells were then shifted to regular growth medium at 37°C and 5% CO_2_ for 1 h. Non-internalized virus was inactivated by a short acid wash for 3 min (40 mM citrate, 135 mM NaCl, 10 mM KCl, pH 3), and the cells were transferred back to regular growth medium. After another 12 h, the cells were fixed with 2% (w/v) glutaraldehyde in 130 mM cacodylate buffer at pH 7.4 containing 2 mM CaCl_2_ and 10 mM MgCl_2_ for 1 h at room temperature. Subsequently the cells were washed and postfixed for 1 h with 1% (w/v) OsO_4_ in 165 mM cacodylate buffer at pH 7.4 containing 1.5% (w/v) K_3_[Fe(CN)_6_], followed by 0.5% (w/v) uranyl acetate in 50% (v/v) ethanol overnight. The cells were embedded in Epon, and 50 nm ultrathin sections were cut parallel to the substrate. Images were taken with an Eagle 4k camera at a Tecnai G2 electron microscope at 200 kV (FEI, Eindhoven, The Netherlands). For quantitation, images were taken at low magnification (6000x) and merged (Adobe Photoshop) to cover the whole cell area. The capsids in the nucleus and in the cytoplasm were counted and the areas of the nucleus and the cytoplasm were measured (ImageJ). Capsid numbers were calculated per area in mm^2^.

## Results

### The N-terminal domain of pUL31 contains a classical bipartite NLS

**Fig 2:**
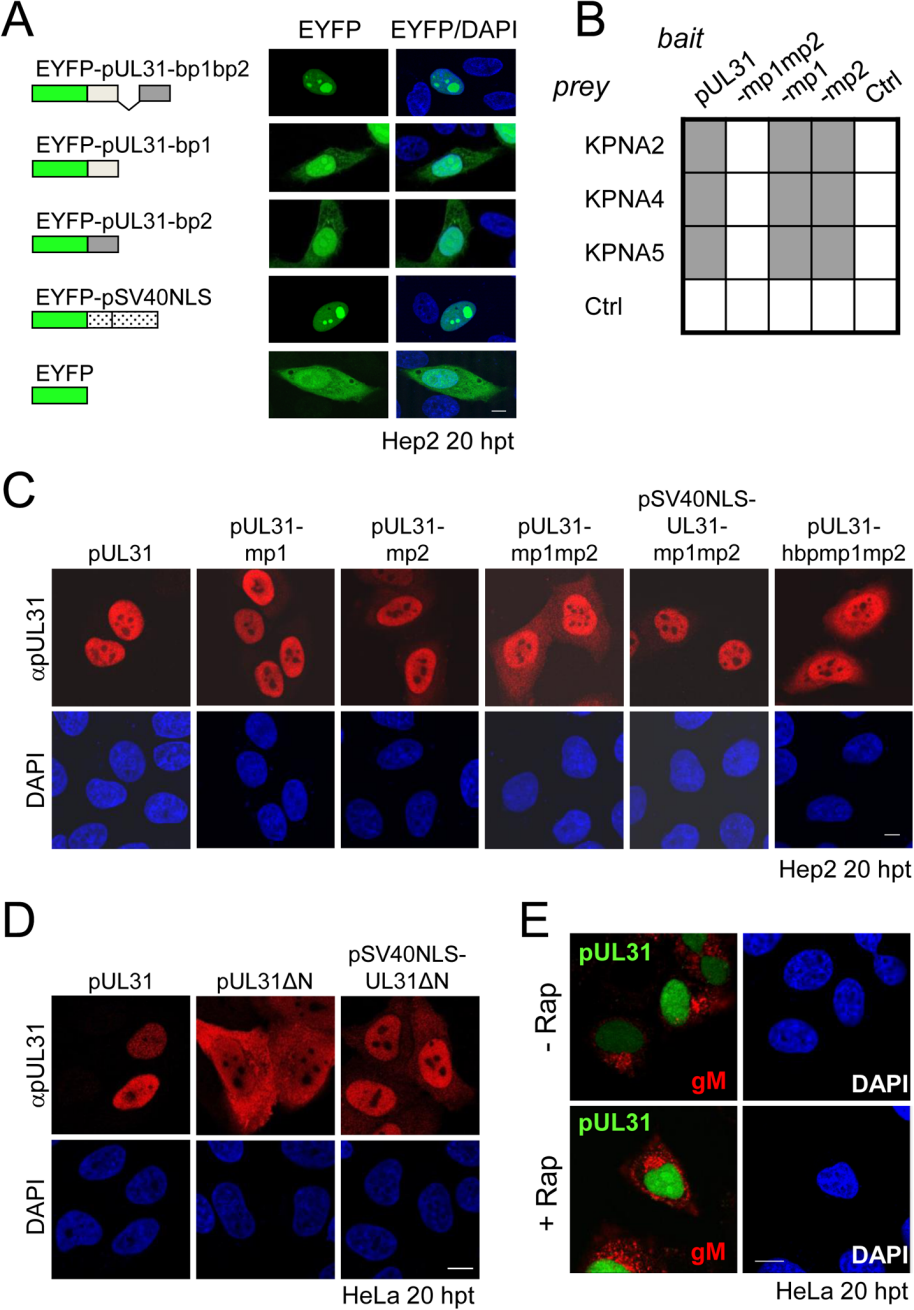
The N-terminal domain of pUL31 contains a classical bipartite NLS. (A) Hep2 cells were transfected with plasmids encoding the EYFP-pUL31-bp1bp2, EYFP-pUL31-bp1, EYFP-pUL31-bp2, or EYFP-SV40NLS fusion proteins; EYFP lacking an NLS was used as negative control. Twenty hours post transfection (hpt), EYFP was detected by confocal fluorescence microscopy. (B) Interaction of pUL31, pUL31-mp1mp2, pUL31-mp1, pUL31-mp2 or a control protein (Ctrl) with α importins KPNA2, KPNA4, or KPNA5 was tested by Y2H using the HIS3 reporter gene activation. Grey squares represent positive and white squares negative results. (C) Hep2 cells were transfected with plasmids encoding myc-tagged pUL31, pUL31-mp1, pUL31-mp2, pUL31-mp1mp2, pSV40NLS-UL31-mp1mp2, or pUL31-hbpmp1mp2, at 20 hpt cells were fixed and analyzed by indirect immunofluorescence microscopy (IF) using primary antibodies directed against the myc-tag on pUL31 and Alexa 594-conjugated secondary antibodies. (D) HeLa cells were transfected with plasmids encoding myc-tagged pUL31, pUL31ΔN, or pSV40NLS-UL31ΔN, at 20 hpt the cells were fixed and analyzed as described in (C). (E) To determine whether pUL31 contains nuclear export activity, the NEX-TRAP assay was applied. HeLa cells were co-transfected with the plasmids pCR3-N-HA-UL10/gM-FKBP and pEYFP-FRB-UL31. Twenty hpt, cells were incubated with anisomycin in absence or presence of rapamycin (-/+ Rap), and processed for IF using anti-gM antibodies followed by Alexa 594-conjugated secondary antibodies, while EYFP was visualized directly. (A, C, D and E) Nuclei were visualized by DAPI, confocal microscopy was applied for analysis. Each scale bar corresponds to 10 μm.

Bioinformatic analysis revealed two patches of positively charged residues composed of RRRSR (basic patch 1; bp1) and RRASRK (basic patch 2; bp2) separated by a linker region within the first 42 residues of HSV-1 pUL31 which resemble a classical bipartite NLS (http://www.expasy.org/; Fig 1A and 1B; [45,50–52]). To be classified as an NLS, a given sequence has to target an unrelated cytoplasmic protein to the nucleus. In addition, it should mediate physical interaction with transport factors of the importin α/β family [62], and its mutagenesis should result in a cytoplasmic localization while re-addition should restore the nuclear residence [62]. EYFP-pUL31-bp1bp2 comprising only residues 21 to 42 of pUL31 (grey in Fig 1B) fused to EYFP was as efficiently targeted to the nucleus as EYFP-SV40NLS (Fig 2A). Both bp1 and bp2 of pUL31 were able to individually target EYFP to the nucleus although less efficiently than the combination of both (Fig 2A) while EYFP alone was located to both cytoplasm and nucleus. Yeast 2-hybrid (Y2H; Fig 2B) and LUMIER experiments (S1A Fig) furthermore demonstrated a physical interaction of pUL31 with transport factors of the importin α family [62]. While pUL31, pUL31-mp1 (mutant patch 1; Fig 1C) as well as pUL31-mp2 (mutant patch 2; Fig 1C) interacted with importins (Fig 2B), pUL31-mp1mp2 did not (Fig 1C; Fig 2B; S1A Fig). Thus, the residues 21 to 42 of pUL31 constitute a classical bipartite NLS that can mediate nuclear import. Its relevance for nuclear import of pUL31 was analyzed by transient expression of myc-tagged pUL31 or mutants thereof (Fig 2C and 2D). pUL31 was exclusively located to the nucleus (Fig 2C and 2D) consistent with previous results [25,35,49,63,64]. Mutant pUL31 with either the first (pUL31-mp1) or the second basic patch (pUL31-mp2) mutated were also located to the nucleus (Fig 2C). pUL31-mp1mp2, with three basic residues in each of the two patches being replaced by neutral residues showed a more pancellular distribution, while adding an SV40NLS to its N-terminus restored its nuclear localization (Fig 2C). An additional exchange of a single residue G10R generated a hyperbasic patch (hbp) identical to residues 21 to 25 (Fig 1C). The resulting pUL31-hbpmp1mp2 was located to both cytoplasm and nucleus, similar to pUL31-mp1mp2 (Fig 2C), indicating that such an artificial basic patch did not rescue nuclear import. pUL31ΔN that lacked the N-terminal 44 residues (Fig 1C) remained cytoplasmic; again its nuclear import was rescued by adding an SV40NLS (Fig 2D; [62]). To reveal any potential export activity of pUL31, we used the NEX-TRAP (nuclear export trapped by rapamycin) assay [58]. EYFP-FRB-pUL31 was exclusively located in the nucleus both in the absence or presence of rapamycin (Fig 2E). pUL31 was unable to reach the cytoplasmic gM-FKBP for rapamycin-induced dimerization at the TGN and thus lacked any export activity (Fig 2E), a finding further corroborated by the interspecies heterokaryon assay [58]. In summary, we conclude that HSV-1 pUL31 harbors an import activity within the N-terminal variable domain, but no export activity. The import activity of pUL31 is composed of a classical bipartite NLS and an unrelated import activity that together mediate the very efficient nuclear import of pUL31.

### The N-terminal domain of pUL31 regulates the interaction with pUL34

**Fig 3:**
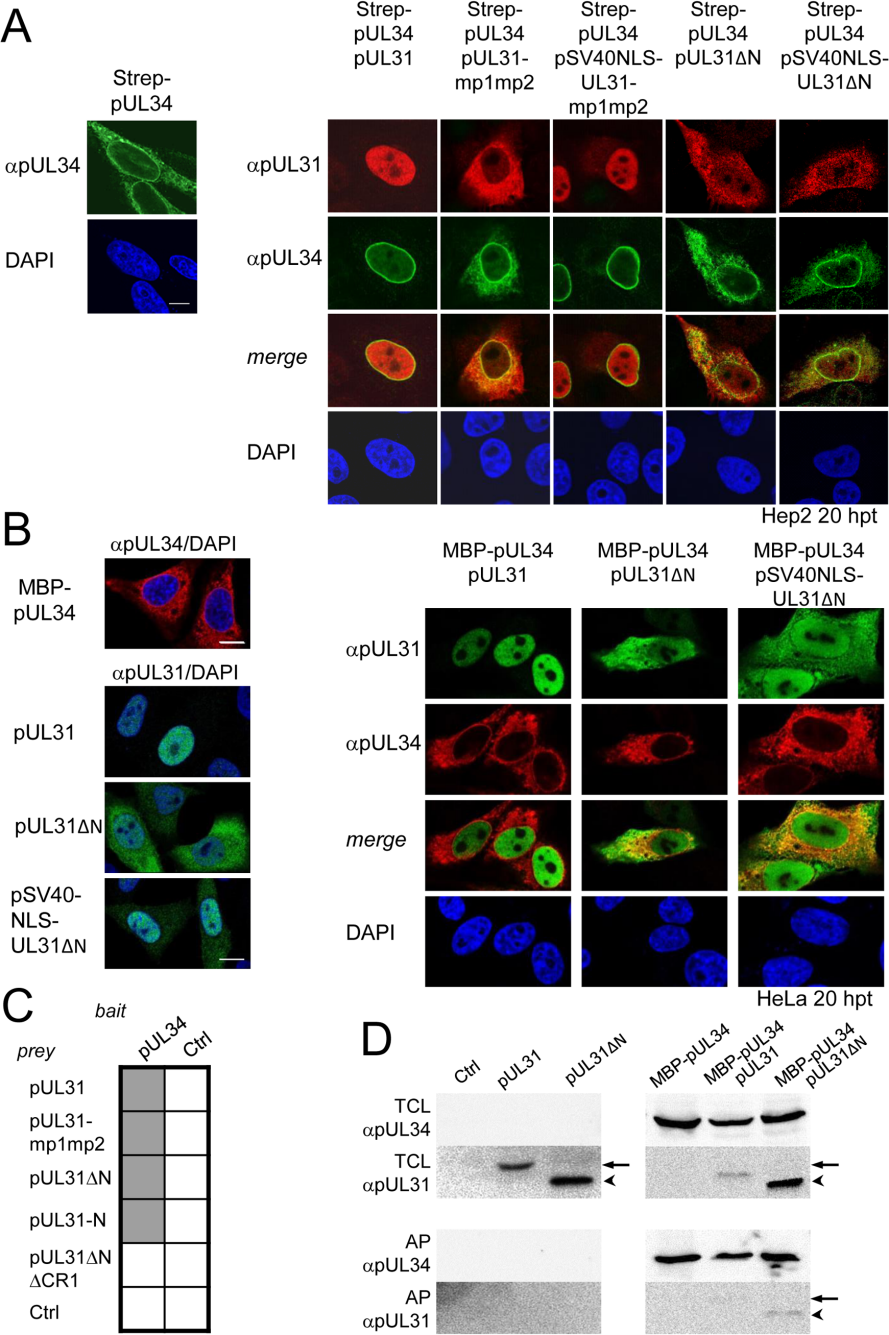
The N-terminal domain of pUL31 regulates interaction with pUL34. (A) To determine the subcellular localization of pUL34 in absence (left) or presence of pUL31, pUL31-mp1mp2, pSV40NLS-UL31-mp1mp2, pUL31ΔN, or pSV40NLS-UL31ΔN (right), Strep-tagged pUL34 was expressed alone or together with myc-tagged pUL31 versions in Hep2 cells. IF analysis was performed at 20 hpt with anti-myc and anti-pUL34 antibodies followed by Alexa 594- and Alexa 488-conjugated antibodies, respectively. (B) Plasmids encoding myc-tagged pUL31, pUL31ΔN, pSV40NLS-UL31ΔN, or MBP-tagged pUL34 were transiently expressed in HeLa cells (left), at 20 hpt IF analysis was performed using anti-myc and anti-MBP antibodies followed by Alexa 488- and Alexa 555-conjugated antibodies, respectively. Furthermore, MBP-pUL34 was co-expressed with myc-tagged pUL31, pUL31ΔN or pSV40NLS-UL31ΔN and analyzed as described before (right). (A, B) Nuclei were visualized by DAPI, for analysis, confocal microscopy was applied. The scale bars correspond to 10 μm. (C) Interaction of pUL34 with pUL31, pUL31-mp1mp2, pUL31ΔN, pUL31-N, pUL31ΔNΔCR1 or a control protein (Ctrl) was tested by Y2H using the HIS3 reporter gene activation. Grey squares represent positive, white squares negative results. (D) To determine the ability of pUL31ΔN to interact with pUL34, HeLa cells were transfected with plasmids encoding MBP-pUL34 and myc-tagged pUL31 or pUL31ΔN (right). For control, cells were transfected with a single plasmid encoding either myc-tagged pUL31 or pUL31ΔN (left). Cells were lysed 24 hpt and processed for affinity-purification using Amylose resin. Total cell lysates (TCL) and affinity-precipitated proteins (AP) were subjected to SDS-PAGE followed by Western blotting using anti-MBP- and anti-myc antibodies followed by peroxidase-conjugated secondary antibodies. pUL31 and pUL31ΔN are indicated by arrows and arrowheads, respectively.

Next we determined the subcellular distribution of the different pUL31 variants in the presence of pUL34. Strep-tagged pUL34 expressed alone was located in cytoplasmic structures and the nuclear envelope (Fig 3A, left; [13,35]). Upon co-expression with pUL31, pUL34 was exclusively targeted to the nuclear envelope while pUL31 was predominantly located in the nucleoplasm (Fig 3A, right) consistent with previous reports [13,35]. Co-expression of pUL31-mp1mp2 and pUL34 resulted in localization of both proteins in the cytoplasm (Fig 3A, right). pSV40NLS-UL31-mp1mp2 co-expressed with pUL34 however was targeted to the nucleoplasm indicating its nuclear import (Fig 3A, right). In contrast, upon co-expression of pUL31ΔN or pSV40NLS-UL31ΔN with pUL34, both proteins were predominantly located in the cytoplasm (Fig 3A, right). Thus, while the addition of an SV40NLS restored nuclear localization of pUL31ΔN in the absence of pUL34 (Fig 2D), this was not the case in the presence of pUL34 (Fig 3A, right).

The nucleoplasmic distribution of pUL31 even upon co-expression with pUL34 (Fig 3A, right) suggested that the interaction of pUL31 and pUL34 might be regulated. pUL34 is a tail-anchored membrane protein while pUL31 *per se* is free to move between cytoplasm and nucleus. The current model for transport of integral membrane proteins to the INM [65] predicts that once anchored in the membrane of the endoplasmic reticulum (ER), pUL34 would be transported laterally along the ER membranes to the outer nuclear membrane (ONM), and the pore membrane (POM), eventually passing the peripheral nuclear pore channels to reach the INM. Transmembrane proteins with cytoplasmic domains above 60 kDa are too large to pass the peripheral nuclear pore channels [65]. To determine the mode of nuclear import of pUL34, pUL34 was fused N-terminally to the maltose-binding protein (MBP) thereby enlarging its cytoplasmic domain to about 60 kDa. Similar to Strep-pUL34 (Fig 3A, left), transiently expressed MBP-pUL34 was targeted to cytoplasmic structures resembling the ER (Fig 3B, left). As shown above, pUL31 expressed alone was exclusively located in the nucleus, pUL31ΔN essentially remained cytoplasmic while pSV40NLS-UL31ΔN was also nuclear (Fig 2D; Fig 3B, left). Upon co-expression of MBP-pUL34 and pUL31, MBP-pUL34 remained cytoplasmic whereas pUL31 was exclusively localized in the nucleus (Fig 3B, right). Thus, MBP-pUL34 was inserted into membranes already in the cytoplasm, but could not enter the nucleus due to its enlarged cytoplasmic domain. In contrast, pUL31 was efficiently imported into the nucleus. Interestingly, a different situation developed upon co-expression of MBP-pUL34 with pUL31ΔN or pSV40NLS-UL31ΔN (Fig 3B, right). With or without an NLS, a considerable amount of either pUL31 protein was retained in the cytoplasm, a finding reminiscent of the results obtained with Strep-pUL34 (Fig 3A, right). This suggested that in the wild type situation, the interaction between pUL34 and pUL31 is prevented in the cytoplasm. In absence of the N-terminal domain however, pUL31ΔN interacted prematurely with pUL34 and/or other components thereby retaining both proteins in the cytoplasm.

To gain further insight into the interaction of pUL31 with pUL34, we used Y2H (Fig 3C) and LUMIER assays (S1B Fig). As expected, pUL31 physically interacted with pUL34 (Fig 3C; S1B Fig). The same was true for pUL31-mp1mp2 and pUL31ΔN (Fig 3C; S1B Fig) consistent with the notion that pUL31-CR1 and potentially other regions of the C-terminal domain contribute to the assembly of the NEC complex [25,26,48,49]. Interestingly, the N-terminal domain of pUL31 also interacted with pUL34, either alone or in co-operation with the neighboring CR1 of pUL31 (Fig 3C). To determine whether pUL31 and pUL31ΔN interacted directly with MBP-pUL34, co-affinity purification was performed. pUL31 or pUL31ΔN were transiently expressed either alone or together with MBP-pUL34. Both myc-pUL31 and myc-pUL31ΔN were co-purified with MBP-pUL34 but not with the Amylose resin alone (Fig 3D). Thus, both proteins had retained the ability to interact with MBP-pUL34 and did so in a specific manner. Interestingly and consistent with previous reports [66], in absence of pUL34 or if spatially separated from it, pUL31 appeared unstable (Fig 3D) while this seemed different with pUL31ΔN (Fig 3D). Taken together, these data show that the NEC proteins pUL34 and pUL31 utilize different transport routes to the nucleus. Most importantly, the presence of a functional N-terminal domain prevents pUL31 from interacting prematurely with pUL34 in the cytoplasm.

### The N-terminal domain of pUL31 harboring basic patches is essential for HSV-1 propagation

**Fig 4:**
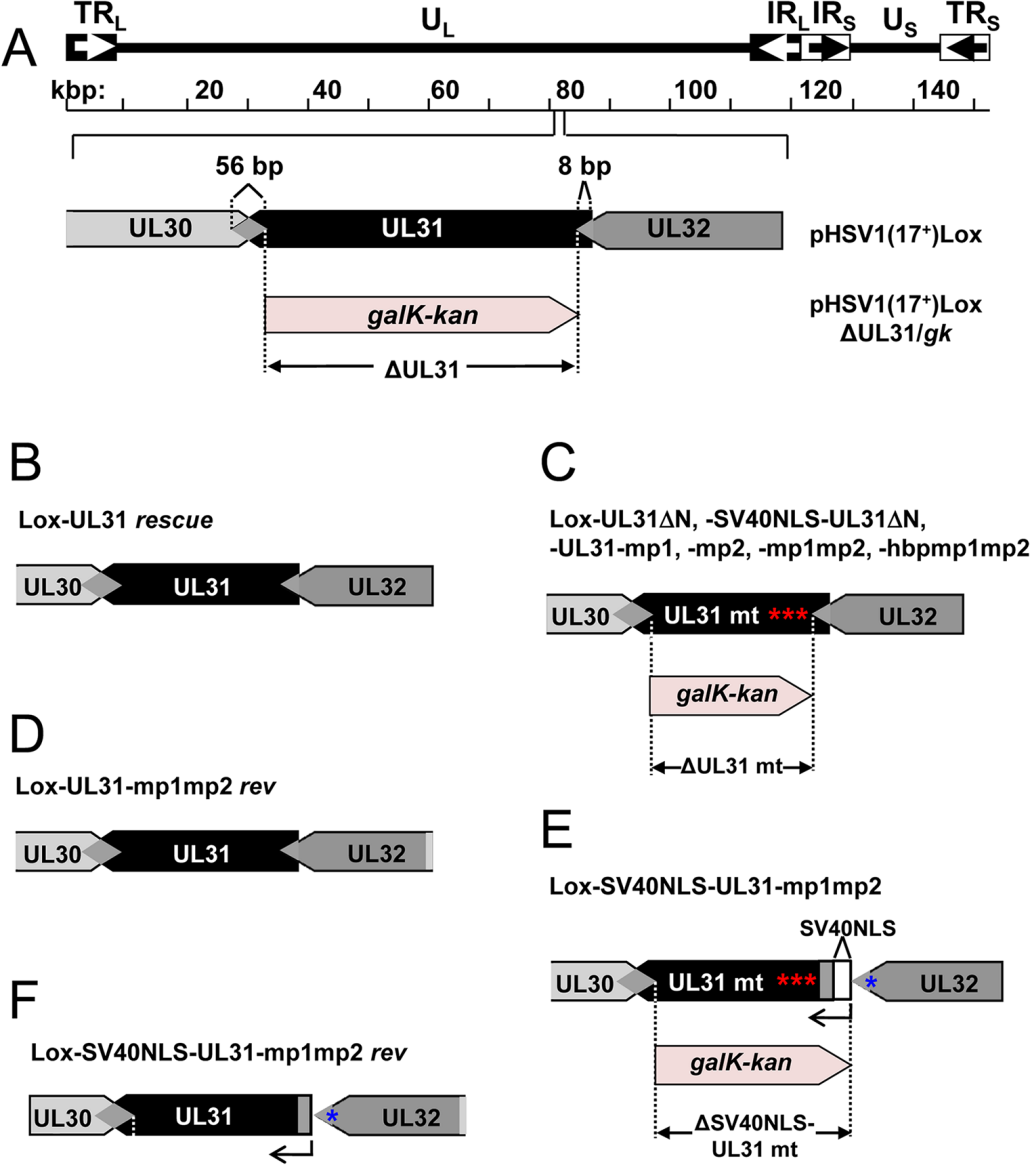
Functional analysis of the N-terminal domain of pUL31. (A) A schematic diagram of the pHSV1(17^+^)Lox genome as well as the strategy to replace the non-overlapping coding region of UL31 (Nucleotides 9 to 865) with the *galK-kan* selection cassette resulting in ΔUL31/*galk* are depicted. (B) To rescue the ΔUL31/*galk* intermediate, the *galK-kan* cassette was replaced by the wt UL31 sequence. (C) To generate the mutants Lox-UL31ΔN, Lox-SV40NLS-UL31ΔN, Lox-UL31-mp1,-mp2,-mp1mp2 and-hbpmp1mp2, the *galK-kan* cassette was replaced by the respective mutant sequences. (D) Lox-UL31-mp1mp2 was reverted to Lox-UL31-mp1mp2 *rev* by a two-step process first replacing the mutant sequence by the *galK-kan* cassette which in turn was replaced by a sequence encoding pUL31. (E) To insert the SV40NLS coding sequence at the 5´end of UL31, the first 8 bp of UL31 overlapping with UL32 were duplicated while at the same time the original start codon of UL31 was mutated without affecting the 3´coding sequence of pUL32. Insertion of the SV40NLS-UL31-mp1mp2 coding sequence led to Lox-SV40NLS-UL31-mp1mp2. (F) Reversion of the mutant described in (E) to a wt situation was performed in a two-step process as described in (D). Note that this revertant carries an 8 bp duplication of the 5´ UL31 region as well as the mutated original start codon of pUL31.

Previous data suggested a role of the N-terminal domain of pUL31 in viral replication [18]. To analyze the function of the pUL31 N-terminal domain in the context of an HSV-1 infection, we generated pHSV1(17^+^)Lox-ΔUL31, Lox-UL31ΔN, Lox-SV40NLS-UL31ΔN, Lox-UL31-mp1, Lox-UL31-mp2, Lox-UL31-mp1mp2, Lox-SV40NLS-UL31-mp1mp2, and Lox-UL31-hbpmp1mp2 using BAC mutagenesis (Fig 4A, 4C and 4E). A rescue mutant was generated for HSV1(17^+^)Lox-ΔUL31/*galK-kan* (Fig 4B), and revertants were made for Lox-UL31-mp1mp2 as well as Lox-SV40NLS-UL31-mp1mp2 resulting in Lox-UL31-mp1mp2 *rev* (Fig 4D) and Lox-SV40NLS-UL31-mp1mp2 *rev* (Fig 4F), respectively. All mutations were verified by restriction digest and sequencing of the mutated regions of the BAC DNAs.

**Fig 5:**
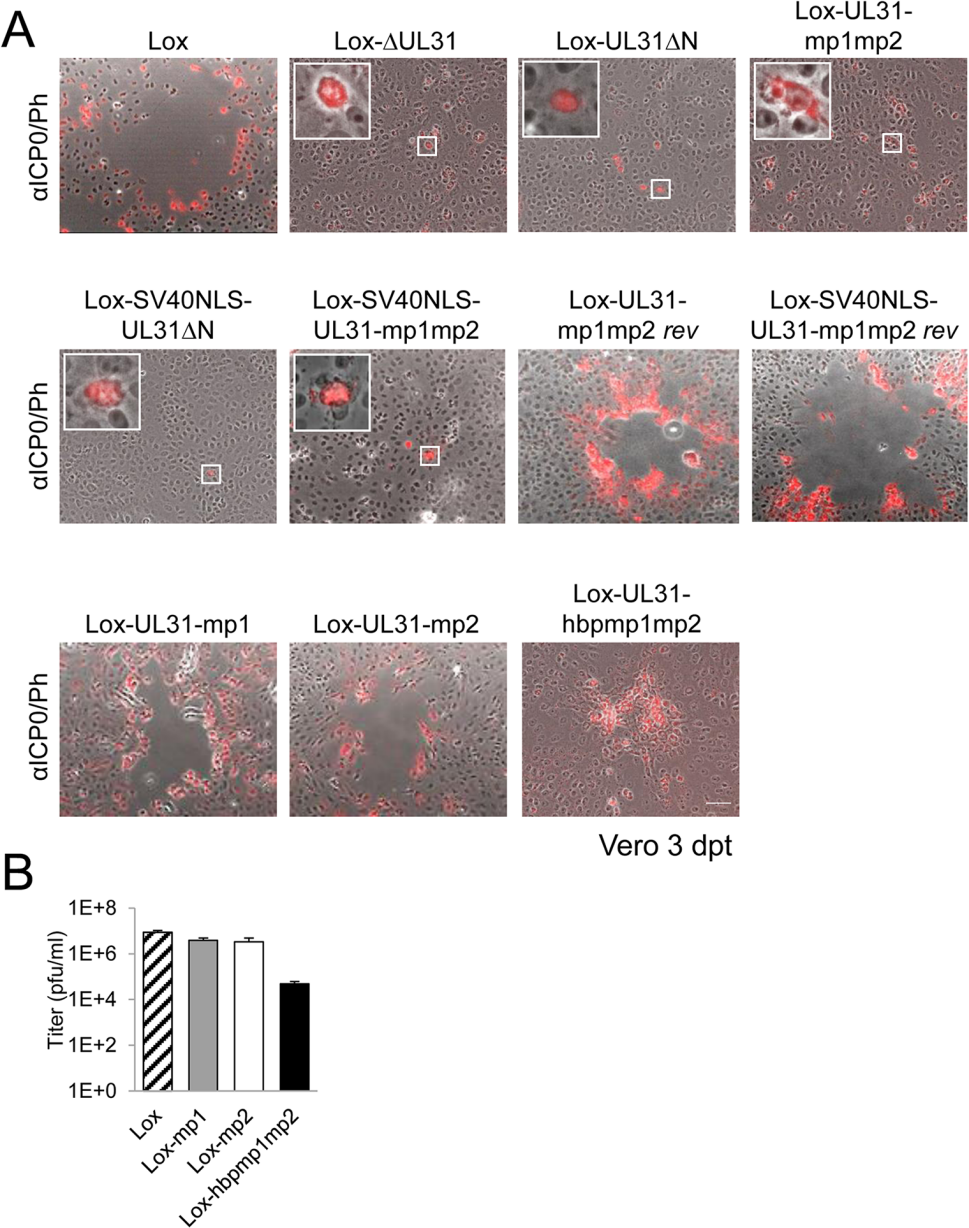
The N-terminal domain of pUL31 harboring basic patches is essential for HSV-1 propagation. (A) The ability of the pHSV1(17^+^)Lox mutants described in Fig 4 to form plaques was tested by transfecting Vero cells with BAC DNA and scoring cytopathic effects at 3 dpt. To visualize transfected cells, IF analysis using anti-ICP0 antibodies followed by Alexa 555-conjugated secondary antibodies was performed. Insets contain magnifications of individual ICP0-positive cells. The scale bar corresponds to 50 μm. (B) To compare the growth properties of mutant viruses HSV1(17^+^)Lox-UL31-mp1, Lox-UL31-mp2, and Lox-UL31-hbpmp1mp2 to the parental virus Lox, Vero cells were infected at an MOI of 0.1, the supernatant was harvested at 48 hpi and titrated on Vero cells in triplicates.

Next, the BAC-DNAs of the respective mutants or the parental pHSV1(17^+^)Lox were transfected into Vero cells (Fig 5A). pHSV1(17^+^)Lox readily formed plaques surrounded by cells expressing the HSV-1 immediate early protein ICP0 (Fig 5A). Consistent with an essential function of HSV-1 pUL31 [15,34,67], transfection of pHSV1(17^+^)Lox-ΔUL31 resulted in single cells expressing ICP0 while no plaques were formed (Fig 5A). Transfection of the pHSV1(17^+^)Lox-UL31ΔN or Lox-UL31-mp1mp2 gave similar results (Fig 5A), and the N-terminal addition of an SV40NLS did not compensate the growth defect of either mutant (Fig 5A). In contrast, the revertants pHSV1(17^+^)Lox-UL31-mp1mp2 *rev* and Lox-SV40NLS-UL31-mp1mp2 *rev* formed plaques as efficiently as the parental strain thus indicating the integrity of the BAC backbone (Fig 5A). These results furthermore demonstrate that the N-terminal addition of the SV40NLS had not impaired pUL31 function. The 3´coding region of the essential UL32 gene overlaps with the 5´coding region of UL31 (Fig 4A; [68]). When Vero cells had been transfected with pHSV1(17^+^)Lox, Lox-ΔUL31, or Lox-UL31-mp1mp2, expression of UL32 was comparable and replication compartments appeared normal as indicated by the subcellular localization of ICP8, the HSV-1 single strand DNA binding protein (S2 Fig; [3,5,8]). Thus, pUL31 mutagenesis had not affected pUL32 expression and function. Nevertheless, the UL31 mutant strains were unable to spread and to form plaques. Together these data show that the N-terminal domain of pUL31 and its basic patches are essential for plaque formation. Most importantly, the addition of the SV40NLS to pUL31-mp1mp2 or pUL31ΔN did not restore plaque formation, suggesting that the N-terminal basic patches of pUL31 convey additional functions beyond merely mediating nuclear import.

### A context-dependent basic patch restores the function of the N-terminal domain of pUL31

To further analyze the role of the N-terminal basic patches the mutants pHSV1(17^+^)Lox-UL31-mp1 or Lox-UL31-mp2 were generated (Fig 1C) and transfected into Vero cells; the resulting plaques were comparable to those of the parental BAC (Fig 5A). Thus, either of the authentic single basic patches was sufficient for virus replication. While the addition of the SV40NLS did not compensate the pUL31-mp1mp2 mutation (Lox-SV40NLS-UL31-mp1mp2 in Fig 5A), Lox-UL31-hbpmp1mp2 with the single mutation G10R that generated a sequence identical to basic patch 1 formed plaques (Fig 5A), although they were considerably smaller than those of the parental strain (Fig 5A). Thus, the G10R exchange partially complemented pUL31-mp1mp2 and restored function. Viral reconstitution showed that Lox-UL31-mp1 and Lox-UL31-mp2 replicated to parental titers, while the titers for Lox-UL31-hbpmp1mp2 were at least 2 logs lower (Fig 5B). Taken together, a single basic patch was sufficient to partially restore the crucial functions harbored within the N-terminal region of pUL31. Since the artificial SV40NLS did not compensate, the relative position within the N-terminal domain and the exact amino acid sequence are apparently important for the essential function of pUL31.

### pUL31 associates with nucleocapsids and requires its N-terminal domain to direct them to sites of primary envelopment

**Fig 6:**
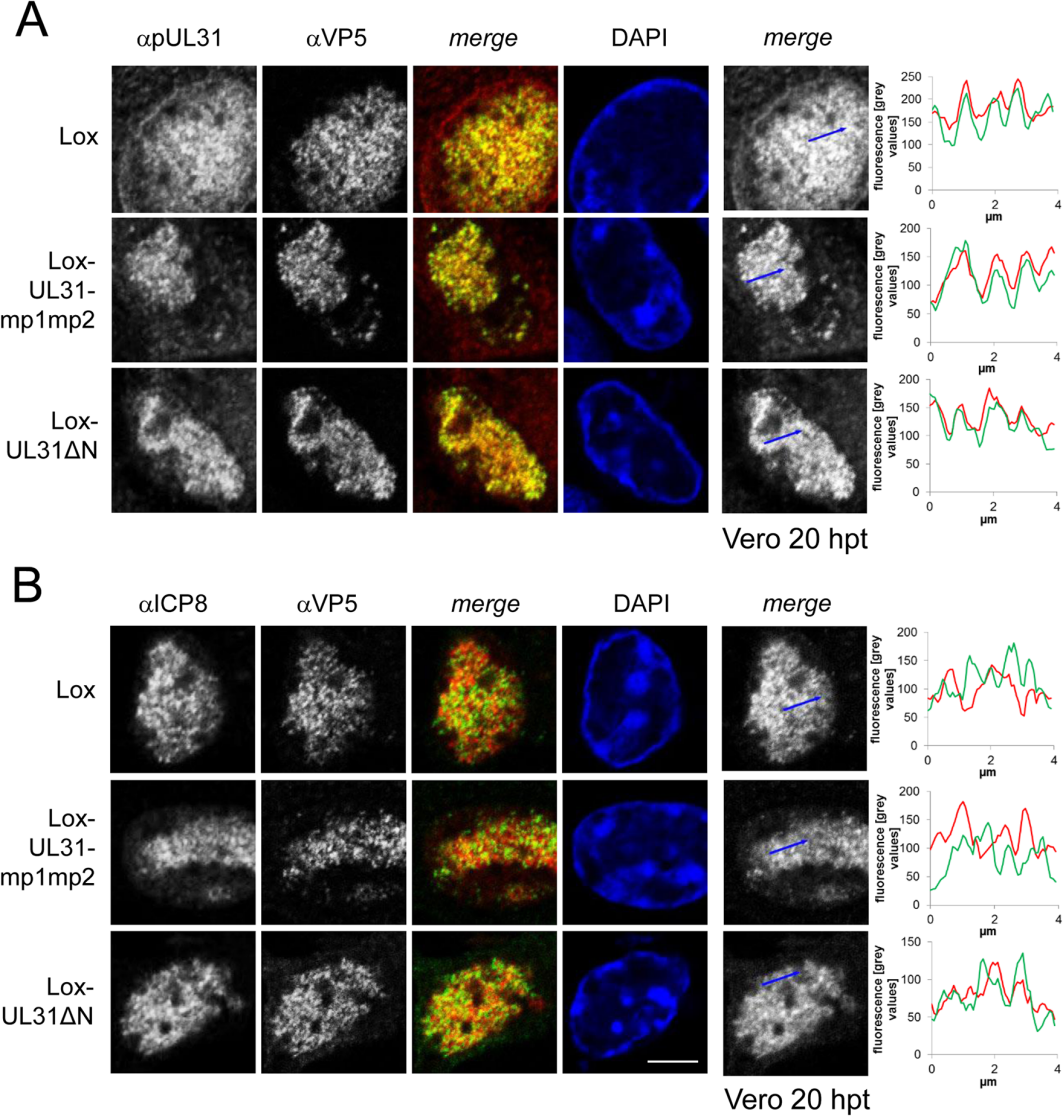
pUL31 associates with nucleocapsids and requires its N-terminal domain to direct them to sites of primary envelopment. To determine the subcellular localization of pUL31 and ICP8 in various UL31 mutant BACs, Vero cells were transfected with BAC DNA of pHSV1(17^+^)Lox, Lox-UL31ΔN or the Lox-UL31-mp1mp2 and analyzed at 20 hpt by IF using monoclonal antibodies directed against VP5 hexons (mAb 8F5) in combination with anti-pUL31 antibodies (A) or anti-ICP8 antibodies (B) followed by Alexa 555- (UL31, ICP8) and Alexa 488- (VP5) conjugated antibodies. Nuclei were visualized by DAPI, and confocal microscopy was applied for analysis. Each scale bar corresponds to 5 μm. To evaluate the degree of co-localization of pUL31 or ICP8 with nucleocapsids, fluorescence signals were measured along the indicated blue arrows and plotted as green (VP5) and red (pUL31, ICP8) values against the length of the measured line using Image J (right panels).

To further decipher the function(s) of the pUL31 N-terminal domain, Vero cells transfected with the parental pHSV1(17^+^)Lox or the mutant BACs (Fig 1C; Tables 1 and 4) were analyzed 20 hours post transfection (hpt) using monoclonal antibodies recognizing mature hexon capsid epitopes (mAb 8F5 [69,70] in combination with antibodies directed against pUL31 ([49]; Fig 6A; S3A Fig) or ICP8 (Fig 6B). Confocal fluorescence microscopy analysis showed that all forms of pUL31 were targeted to the nucleoplasm (Fig 6A; S3A Fig). Thus, during HSV-1 infection, both the N-terminal authentic and the SV40NLS were dispensable for nuclear targeting of pUL31. There was no labeling in Vero cells transfected with Lox-ΔUL31 demonstrating the specificity of the anti-pUL31 antibodies (S3A Fig). After transfection with parental pHSV1(17^+^)Lox, Lox-UL31ΔN or Lox-UL31-mp1mp2 (Fig 6A), the subnuclear localization of pUL31 appeared punctuate and correlated with the capsid protein VP5 detected by antibodies to mature hexon epitopes (Fig 6A; [69]). While wt pUL31 was located to both nucleoplasm and nuclear envelope, pUL31ΔN or pUL31-mp1mp2 co-localized with capsids in the nucleoplasm, but not with the nuclear rim (Fig 6A). In contrast, ICP8, a marker of replication compartments [8], had a different subnuclear localization than the capsids (Fig 6B). Line histograms revealed that pUL31 and capsids largely co-localized while this was not the case for ICP8 and capsids (Fig 6A and 6B, right panels). Thus, pUL31, pUL31ΔN and pUL31-mp1mp2 could associate with capsids. Upon transfection with pHSV1(17^+^)Lox-UL31ΔN or Lox-SV40NLS-UL31ΔN, pUL31ΔN or SV40NLS-UL31ΔN were partially retained in the cytoplasm (S4A Fig), reminiscent of their localization after co-expression of pUL31ΔN or pSV40NLS-UL31ΔN with pUL34 (Fig 3A and 3B). Thus, the absence of the amino-terminal domain conferred partial cytoplasmic retention of pUL31 while nuclear import and targeting to capsids still occurred. Taken together, pUL31 was targeted to capsids present in the nucleoplasm and the C-terminal domain of pUL31 was sufficient to mediate this association.

To analyze the subcellular distribution of pUL34 upon mutagenesis of the N-terminal domain of pUL31, Vero cells were transfected with pHSV1(17^+^)Lox or the UL31 BAC mutants (S3B Fig; S4B Fig). As expected, in cells transfected with the parental Lox, pUL34 was located to the nuclear envelope. In cells transfected with Lox-ΔUL31, Lox-UL31-mp1mp2, Lox-UL31ΔN, or Lox-SV40NLS-UL31ΔN, pUL34 was also targeted to the nuclear envelope although its distribution seemed more patchy (S3B Fig; S4B Fig). This suggested that in addition to pUL31, other viral and host factors contribute to targeting of pUL34 to the nuclear envelope, a finding also supported by other recent reports [9,33,34].

### Nucleocapsids of HSV1(17^+^)Lox-UL31-hbpmp1mp2 are impaired in nuclear egress

**Fig 7:**
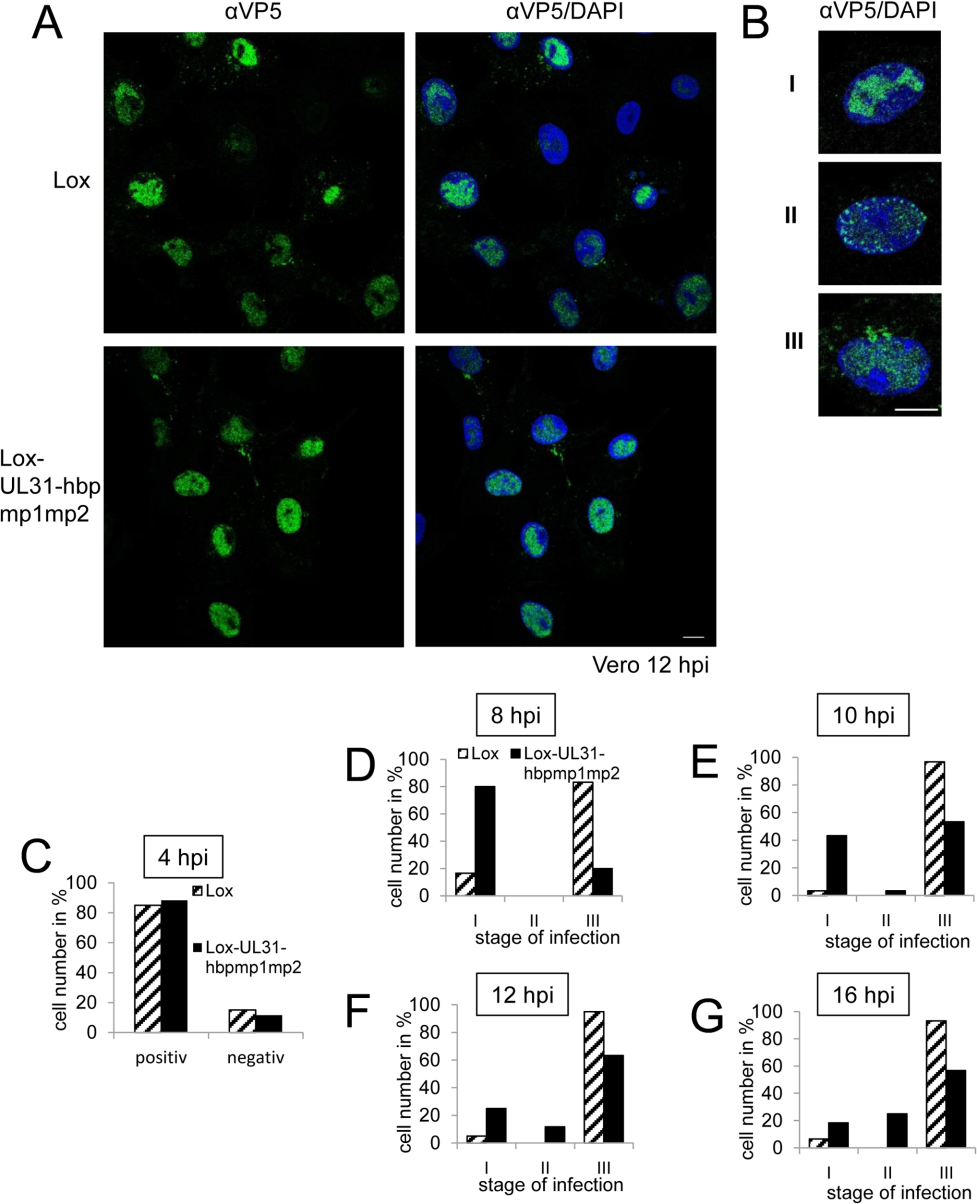
Nucleocapsids of HSV1(17^+^)Lox-UL31-hbpmp1mp2 are impaired in nuclear egress. (A, B; D-G) To analyze the subcellular localization of nucleocapsids, Vero cells were infected at an MOI of 1 with HSV1(17^+^)Lox or Lox-UL31-hbpmp1mp2, fixed at various timepoints and analyzed by IF using monoclonal antibodies directed against VP5 hexons (mAb 8F5) followed by secondary reagents. Nuclei were visualized by DAPI. (A) Overview of the different stages of infection with regard to the nuclear and cytoplasmic distribution of nucleocapsids. (B) Examples of the three phenotypes of infection: Categories I and II with capsids condensed in the center of the nucleus (I), or exclusively located in the nucleus, dispersed but also peripherally speckled (II). Cells with capsids in the nucleus as well as in the cytoplasm are defined as category III. (C) Vero cells were infected at an MOI of 1 with HSV1(17^+^)Lox or Lox-UL31-hbpmp1mp2, fixed at 4 hpi and analyzed by IF using antibodies against ICP0 followed by secondary antibodies. (D-G) To analyze nuclear capsid egress, Vero cells were infected for 8 hpi (D), 10 hpi (E), 12 hpi (F), or 16 hpi (G) and analyzed as described above. The infection rate and the frequency distribution of the three stages were quantified by classifying 60 cells per condition. The scale bar corresponds to 10 μm. For analysis, confocal microscopy was applied.

A single amino-acid exchange within pUL31-mp1mp2 resulting in pUL31-hbpmp1mp2 rescued the functions of the N-terminal domain (Fig 5B). To further define these functions, Vero cells were infected with HSV1(17^+^)Lox or Lox-UL31-hbpmp1mp2 at an MOI of 1 (Fig 7A–7G). About 80% of the cells infected with either HSV1(17^+^)Lox or Lox-UL31-hbpmp1mp2 expressed ICP0 at 4 hours post infection (hpi) (Fig 7C). Nevertheless, three phenotypes could be distinguished (Fig 7A and 7B): cells with capsids condensed in the nucleoplasm (category I), cells devoid of cytoplasmic capsids but with a dispersed and speckled appearance of nuclear capsids (category II), and cells with both nuclear and cytoplasmic capsids (category III). To quantify these phenotypes, cells were analyzed at 8 hpi (Fig 7D), 10 hpi (Fig 7E), 12 hpi (Fig 7A, 7B and 7F) or 16 hpi (Fig 7G) with a total of 60 infected cells for each condition (Fig 7D–7G). In the majority of cells infected with the parental strain, the nucleocapsids were dispersed throughout the nucleus with a considerable number of cytoplasmic capsids already at 8 hpi (Fig 7D). Only a few cells fell into category I or II while category III dominated, and this phenotype was further enhanced at later time points. In contrast, upon infection with Lox-UL31-hbpmp1mp2, the majority of cells belonged to category I at 8 hpi (Fig 7D). At 10 and 12 hpi, cytoplasmic capsids were detected in about 50 and 60%, respectively, of the cells (category III; Fig 7E and 7F). At 16 hpi, the percentage of cells in category III remained rather constant, at the same time, nuclei containing dispersed capsids (category II) increased, a phenotype rarely observed with the parental virus (Fig 7G).

**Fig 8:**
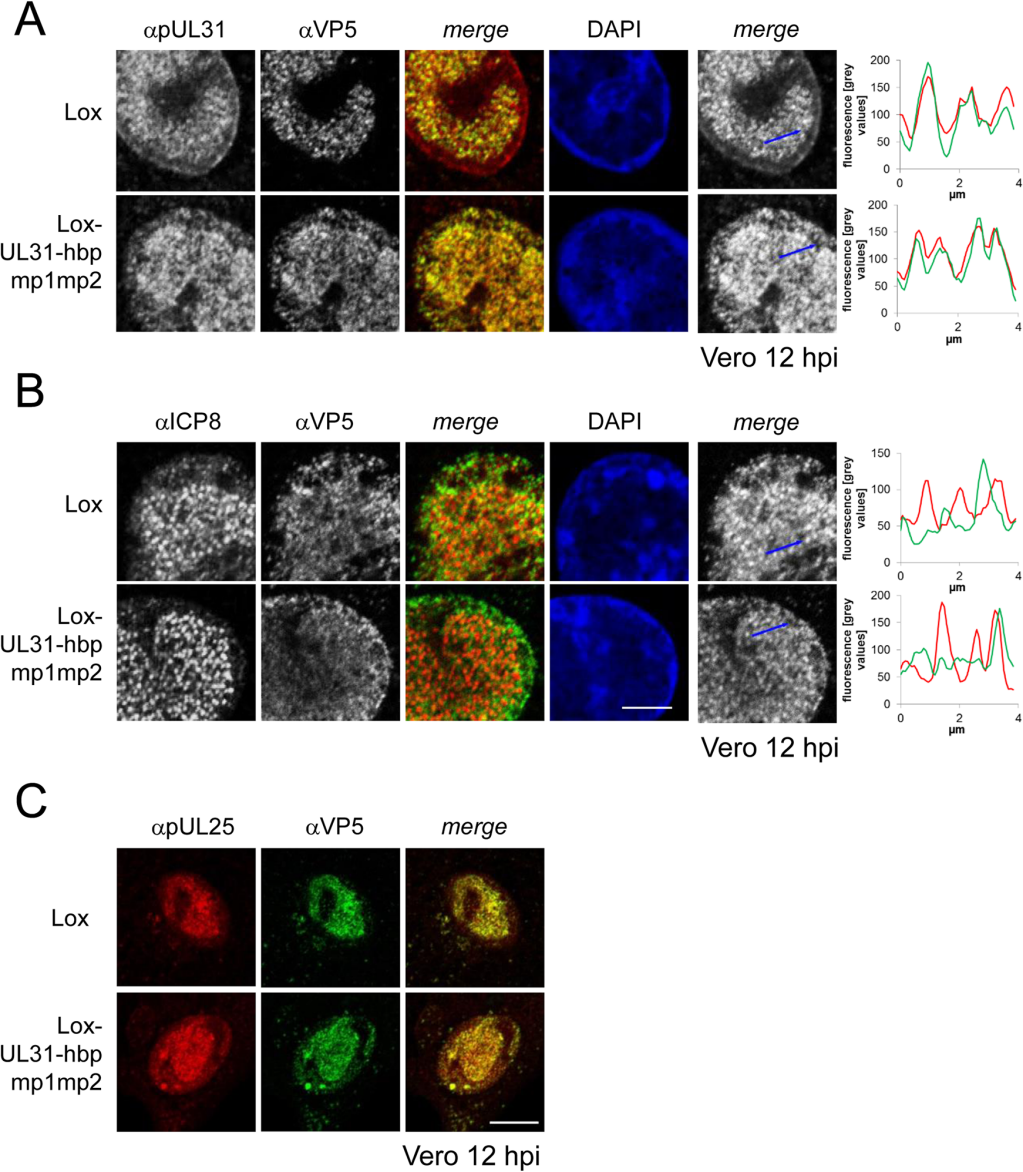
Detailed analysis of Lox-UL31-hbpmp1mp2. To analyze the subcellular localization of nucleocapsids, Vero cells were infected with HSV1(17^+^)Lox or Lox-UL31-hbpmp1mp2 using an MOI of 1 and analyzed at 12 hpi by IF using antibodies against VP5 (mAb 8F5) in combination with anti-pUL31 antibodies (A) or anti-ICP8 antibodies (B) followed by Alexa 555- (pUL31, ICP8) and Alexa 488- (VP5) conjugated secondary antibodies. To evaluate the degree of co-localization, fluorescence signals were measured along the indicated blue arrows and plotted as green (VP5) and red (pUL31, ICP8) values against the length of the measured line using Image J (right). The scale bar corresponds to 5 μm. (C) To determine the subcellular localization of VP5 and pUL25, Vero cells were infected at an MOI of 1 with HSV1(17^+^)Lox or Lox-UL31-hbpmp1mp2 and analyzed at 12 hpi by IF using anti-VP5 and anti-pUL25 antibodies followed by secondary antibodies. The scale bar corresponds to 10 μm. For analysis, confocal microscopy was applied.

For closer inspection, Vero cells infected with the parental virus were compared to cells infected with Lox-UL31-hbpmp1mp2 (Fig 8A and 8B; S5 Fig). pUL31-hbpmp1mp2 had also been targeted to nucleocapsids (Fig 8A), whereas the nuclear replication compartments containing ICP8 did not co-localize with the nuclear sites of capsid assembly (Fig 8B). Line histograms clearly showed that the capsid protein VP5 co-localized well with pUL31-hbpmp1mp2 but not with ICP8 (Fig 8A and 8B, right panels). Depending on the stage of infection, pUL31-hbpmp1mp2 co-localized with capsids enriched in speckles in close association with the nuclear envelope (Fig 7A and 7B; S5 Fig). However, unlike in cells infected with the parental virus, a clear nuclear rim localization of pUL31 could not be detected [38,39]. The pattern of the subcellular localization of pUL25, a minor-capsid associated protein, was very similar to that of the major capsid protein VP5 and also co-localized with both wt pUL31 and pUL31-hbpmp1mp2 (Fig 8C). Upon infection with Lox-UL31-hbpmp1mp2, pUL34 was as efficiently targeted to the nuclear envelope as with the parental Lox (S6 Fig). To summarize, pUL31 was associated with nucleocapsids that had recruited pUL25 and escorted them to the nuclear periphery, a process that was delayed for Lox-UL31-hbpmp1mp2 concomitant with a defect in viral replication. Thus, basic patches within the N-terminal domain of pUL31 are required for efficient translocation of capsids from the nucleoplasm and to the sites of primary envelopment at the nuclear envelope.

### pUL31-hbpmp1mp2 forms mature nucleocapsids delayed in nuclear egress

**Fig 9:**
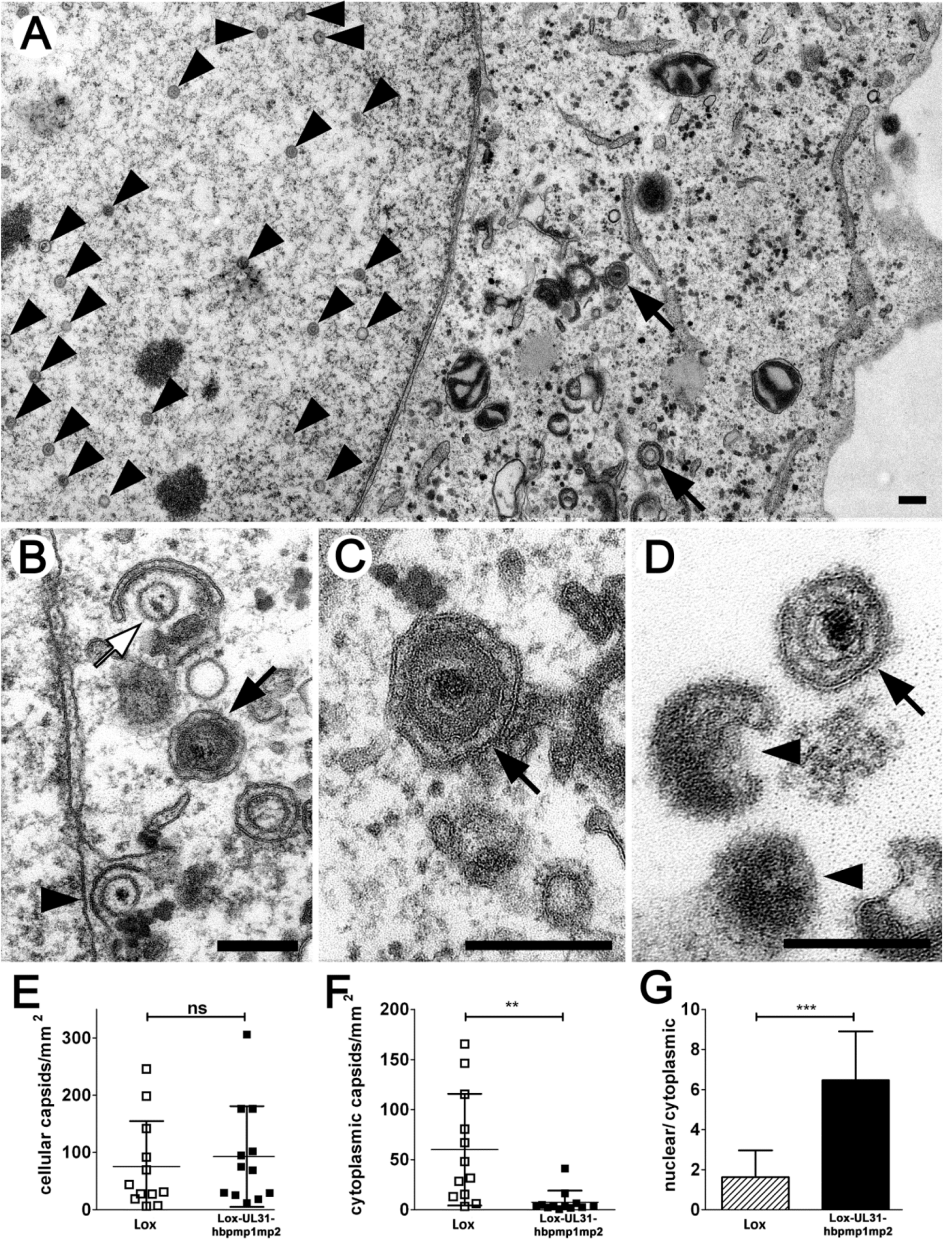
pUL31-hbpmp1mp2 forms mature nucleocapsids delayed in nuclear egress. Vero cells were infected with HSV1(17^+^)Lox or Lox-UL31-hbpmp1mp2 at an MOI of 1, fixed at 13 or 17 hpi and analyzed by electron microscopy. (A-D) Electron micrographs of Lox-UL31-hbpmp1mp2 at 13 hpi revealed all intracellular stages of virus maturation: capsids in the nuclear matrix (A, arrowheads), capsids traversing the nuclear envelope (B, arrowhead), early stages of secondary envelopment (B, white arrow), and fully enveloped virions in vesicles (A-C, black arrows). At the extracellular surface of the cells, virions (D, black arrow) and L-particles (D, arrowheads) were present. (E-G) Quantitation of the number of capsids in the nucleus and the cytoplasm after infection with HSV1(17^+^)Lox or Lox-UL31-hbpmp1mp2 for 17 hpi; (E) number of intracellular capsids, (F) number of cytoplasmic capsids, (G) ratio of nuclear to total cellular capsids. 12 cells of each condition were analyzed. (E, F) Each data point corresponds to one cell. (G) The ratio of the number of nuclear capsids per mm² and the number of cellular capsids per mm² was calculated and expressed as arithmetic means. (E-G) SDs are indicated. Significance was calculated with an unpaired t test using the software Graphpad Prism (ns = not significant, p = 0.6196; **, p = 0.0043; ***. p<0.0001). Scale bars equal 200 nm.

For high-resolution analysis, Vero cells were infected with Lox-UL31-hbpmp1mp2, fixed at 13 hpi (Fig 9) and analyzed by electron microscopy. Essentially all intracellular stages of virus maturation had been formed. These included mature capsids in the nuclear matrix (Fig 9A, arrowhead), capsids traversing the nuclear envelope (Fig 9B, arrowhead), early stages of secondary envelopment (Fig 9B, white arrow), and fully enveloped virions in vesicles (Fig 9A–9C, black arrows). Fully matured virions (Fig 9D, black arrow) as well as L-particles (Fig 9D, arrowheads) were located at the extracellular surface of the cells consistent with the production of infectious particles. Quantitation of the number of capsids in the nucleus and the cytoplasm revealed that although the cells contained similar amounts of capsids as after infection with the parental HSV1(17^+^)Lox (Fig 9E), the number of cytoplasmic virus particles was significantly reduced after infection with Lox-UL31-hbpmp1mp2 (Fig 9F). Hence the ratio of nuclear to cytoplasmic capsids was significantly increased in cells infected with the mutant (Fig 9G). We thus conclude that essentially all steps of viral morphogenesis occurred in cells infected with the Lox-UL31-hbpmp1mp2. To summarize, HSV1(17^+^)Lox-UL31-hbpmp1mp2 formed mature capsids, and pUL31-hbpmp1mp2 co-localized with mature VP5 hexon epitopes and pUL25. However, the escort of the capsids to the nuclear envelope seemed to be delayed for HSV1(17^+^)Lox-UL31-hbpmp1mp2, consistent with a reduced production of infectious virions. Thus, the N-terminal basic patches of pUL31 were required for efficient translocation of capsids from the nucleoplasm to sites of primary envelopment.

## Discussion

The NEC of the herpesviruses is composed of two conserved essential proteins, called pUL34 and pUL31 in HSV-1, that are required for primary envelopment at the INM and for nuclear egress of newly formed capsids [10,11]. *In vivo*, their co-expression leads to the formation of empty vesicles in the perinuclear space [28,29]. *In vitro* systems recently revealed that these two proteins represent the minimal virus-encoded membrane-budding machinery that contains intrinsic activity to drive budding and scission of membrane vesicles [30,31]. The observation that a complex formed between pUL34 and pUL31 exhibits membrane budding activity [30], instantly implies that the NEC activity needs to be spatially and temporally controlled and confined to sites of nuclear egress to enable efficient capsid nuclear egress and at the same time prevent perturbations on cytoplasmic membranes.

This study shows that pUL34 and pUL31 utilized separate routes to the nucleus. pUL31 enters the nucleus through the central nuclear pore channels. The N-terminal domain of HSV-1 pUL31 contains a classical bipartite NLS composed of two basic patches that bound importin α and mediated nuclear import congruent with data on the pUL31 orthologs of HSV-2 [51], murine cytomegalovirus (MCMV; [45]), human cytomegalovirus (HCMV; [52]), and pseudorabies virus (PrV; [50]). The bipartite NLS of HSV-1 pUL31, however, was not essential for nuclear import as pUL31-mp1mp2 was also imported into the nucleus, both in absence and presence of other HSV-1 proteins, consistent with results obtained for pUL31 of HSV-2 [51]. Thus, auxiliary modes of nuclear import are likely to exist, in form of additional non-classical import sequences embedded in the amino-terminal domain [51] and likely also by piggy-backing with other viral partners, for example the pUL17/pUL25 complex [38,39,41,71] or ICP22 [34].

In contrast to soluble proteins like pUL31, integral membrane proteins such as pUL34 need to traverse the nuclear pore through its peripheral channels [65] that physically restrict the size of the cyto-/nucleoplasmically exposed domain [65]. pUL31 and pUL34 form a complex of about 60 kDa [30] that, if formed prior to nuclear import, would be too close to the size limitations of the peripheral nuclear pore channels [65]. Thus, a mechanism is required to prevent premature cytoplasmic association of pUL34 and pUL31. Data presented in this study show that pUL34 with its N-terminal domain enlarged by attaching MBP to mimic the size of a pUL34/pUL31 complex, was retained in the cytoplasm, while co-expressed pUL31 was still imported into the nucleus. Recent analysis of the HCMV NEC proteins also shows that pUL53, the ortholog of pUL31, precedes pUL50, the ortholog of pUL34, in nuclear import [52]. Together we conclude that the interaction of pUL31 and pUL34 and their orthologs is prevented in the cytoplasm to allow for their independent import into the nucleus.

Strikingly, we observed that in absence of the 44 N-terminal residues of pUL31, both pUL31 and pUL34 were retained in the cytoplasm, and that the addition of the SV40NLS to pUL31ΔN was unable to confer its nuclear import in the presence of pUL34. Furthermore, we show that pUL31ΔN physically interacted with pUL34, and that both proteins co-localized in the cytoplasm consistent with earlier studies [25,48]. Together these data indicate that pUL31ΔN was retained in the cytoplasm most likely by an interaction with membrane-associated pUL34. A NEC complex preformed and retained in the cytoplasm would be unavailable for its essential nuclear functions. In addition, premature NEC formation in the cytoplasm would unleash the intrinsic activity of the NEC to drive budding and scission of membranes [30] with deleterious effects on membrane integrity and function. Our results here suggest that the N-terminal domain of pUL31 provides a mechanism to prevent premature cytoplasmic NEC activity, and that it is critical in modulating the interaction of pUL31 with pUL34.

While pUL31ΔN and pSV40NLS-UL31ΔN were partially retained in the cytoplasm, significant amounts of them nevertheless had reached the nucleoplasm, most likely because they were expressed prior to pUL34. Despite the nuclear import of these pUL31 variants, there was a drastic reduction in replication for Lox-UL31ΔN and Lox-SV40NLS-UL31ΔN, implying that neither pSV40NLS-UL31ΔN nor pUL31ΔN were able to support capsid nuclear egress. Thus other functions than the NLS are encoded by the N-terminal domain of pUL31 and critical for viral propagation.

We show that once imported into the nucleoplasm, pUL31 was primarily targeted to nucleocapsids consistent with previous reports [35,39,41]. While the nuclear capsids of Lox-UL31ΔN and Lox-UL31-mp1mp2 had recruited the mutated pUL31 proteins, plaque formation was still inhibited. Obviously, the C-terminal domain of pUL31 was sufficient to bind pUL31 to capsids whereas the N-terminal basic patches must contribute essential functions downstream of this event. The mutation G10R generated an artificial basic patch in pUL31-hbpmp1mp2 that partially restored its function: capsids translocated to the nuclear periphery although with lower efficiency than in the parental virus. Thus, a single N-terminal basic patch was critical in promoting capsid translocation and nuclear egress. This mutant thus unveiled a previously unanticipated sequence of events where pUL31 initially interacts with capsids at their assembly sites and then escorts them to the nuclear periphery, a process that is obscured during the fast progression of a natural HSV-1 infection. The basic patches may promote conformational changes of pUL31 and serve as a platform to recruit hitherto unknown viral and/or host proteins that rearrange the host chromatin and mediate capsid transport through the nucleoplasm to the INM [3,5]. Thereby, the capsids may be dispersed and translocated to sites of primary envelopment, as observed in this study and supported by previous findings [3].

The subnuclear localization of HSV-1 pUL31 correlated well with that of capsids harboring mature VP5 hexon epitopes and the minor-capsid associated protein pUL25, a finding further supported by biochemical evidence [38,39,41]. Thus, pUL25 and pUL17 that together form the capsid vertex-specific complex (CVSC) and promote cleavage and packaging of viral genomes into capsids [10,11], could link pUL31 with nucleocapsids to cooperate in maturation [15]. In such a scenario, the C-terminal domain of pUL31 composed of CR1-4 and sufficient for capsid binding may contribute to capsid maturation [38,39,41]. Interestingly, CR2 and CR4 of M53, the MCMV ortholog of pUL31 are also involved in DNA genome cleavage/packaging [37,40]. Furthermore, pUL31 physically interacts with a pUL25/pUL17 complex even in the absence of capsids [38]. Thus, a pre-formed complex of pUL31 and pUL25/pUL17 may bind to capsids during DNA cleavage and packaging. By such a mechanism, the capsid-associated pUL31 may contribute to completion of genome packaging [15,36–41] and selection for primary envelopment at the INM ([10,11], and references therein).

The domains of pUL31 that interact with pUL34 seem to be initially masked but must eventually be exposed to drive NEC formation and activity. Upon infection with Lox-UL31-hbpmp1mp2, capsids with mature VP5 epitopes accumulated in close vicinity to the INM suggesting that primary envelopment was compromised if the authentic basic patches had been neutralized. Thus, the basic patches could serve two functions; to trigger the translocation of capsids from the nucleoplasm to the nuclear envelope, and to promote budding, but only in the presence of capsids. Indeed, *in vitro* data support an active role of the pUL31 basic patches in regulating the budding process; their deletion abrogated NEC activity but not the formation of the complex and its association with membranes [30]. Interestingly, cytoplasmic membranes harboring pUL34 and pUL31ΔN appeared undisturbed suggesting that a pUL31ΔN/pUL34 complex lacks NEC activity. An attractive scenario suggests that the basic patches in the N-terminal domain of pUL31 promote membrane budding by stabilizing a conformational switch within pUL34. In contrast, the non-essential US3 protein kinase seems not to be critical although it phosphorylates several sites within the N-terminal domain of pUL31 [18]. The severe phenotype of Lox-UL31ΔN and Lox-UL31-mp1mp2 contrasts that of HSV-1 strains lacking US3 [19,32] suggesting that pUL31 phosphorylation has modulatory or cell-type specific effects.

We and others have shown that several regions of HSV-1 pUL31 interact with pUL34. The CR1 of all pUL31 orthologs interacts with pUL34 [45–47] probably involving pUL34 residues 137 to 181 [25,26,49,67]. As an extension of pUL31-CR1, the N-terminal domain could contribute to the interaction with pUL34. Furthermore, the C-terminal domain of pUL31 seems to be functionally linked to the N-terminal domain of pUL34 [25,48,50]. Upon infection with Lox-UL31-hbpmp1mp2, we observed capsid speckles with mature hexon epitopes that accumulated in close vicinity to pUL34 at the INM. These speckles resembled the phenotype of a C-terminal mutant of M53, the pUL31 ortholog of MCMV, that is also impaired in capsid egress [37] suggesting that the N- and C-terminal domains of pUL31 cooperate to coordinate the interaction with pUL34. Upon association with capsids in the nucleoplasm, pUL31 may undergo a conformational switch from a closed conformation with the N- and potentially the C-terminal domain covering pUL34-interacting regions to an open conformation that allows for interaction with pUL34 and thus capsid docking and budding at the INM. The interaction between pUL31 and pUL34 may even involve multiple and sequential conformational changes established during NEC formation and membrane budding, a process that appears to be highly orchestrated [22–27]. Our understanding of the precise molecular mechanism however must await the structural resolution of the NEC.

**Fig 10:**
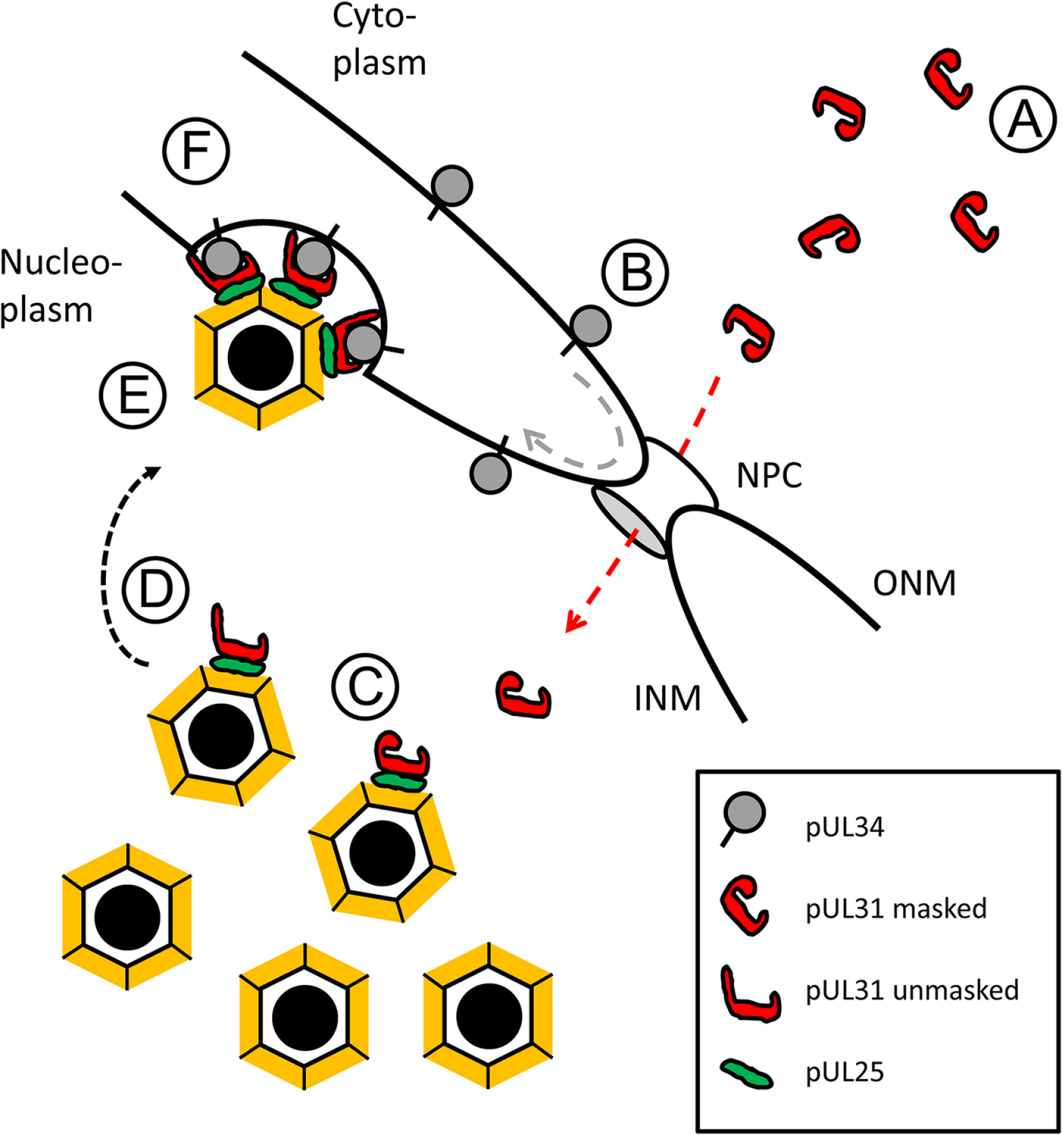
HSV-1 pUL31-mediated escort of capsids to the nuclear periphery. A schematic representation of the route, pUL31 takes after its synthesis from the cytoplasm to nucleocapsids to escort them from the sites of assembly to the sites of primary envelopment at the inner nuclear membrane (INM): A) Cytoplasmic pUL31 is masked and inhibited by adopting a conformation that prevents a premature interaction with pUL34. B) pUL31 (red dashed line) is imported into the nucleus independently of pUL34 (grey dashed line). C) In the nucleus, pUL31 associates with capsids at the sites of their assembly and potentially assists in genome packaging. D) Conformational changes in its N-terminal domain unmask pUL31 and target it to sites of primary envelopment. E) Capsid-associated pUL31 interacts with pUL34 to form the NEC. F) A series of sequential interactions between pUL31 and pUL34 molecules initiates membrane wrapping of the capsid until primary envelopment is complete. The nuclear pore complex (NPC), the outer nuclear membrane (ONM) and the inner nuclear membrane (INM) are depicted.

Our data together with those of others suggest a highly regulated sequence of events during primary envelopment of HSV-1 (Fig 10; [10,11,25–27,35,38,39,41,44]: (A) Newly synthesized, cytoplasmic pUL31 is masked and inhibited by adopting a conformation that prevents premature interaction with pUL34, (B) pUL31 is imported into the nucleus independently of pUL34, but possibly in a complex with other viral and host proteins, (C) pUL31 associates with nucleocapsids and potentially contributes to genome packaging, (D) conformational changes in its N-terminal domain contribute to targeting of mature capsids to the INM, (E) capsid-associated pUL31 interacts with membrane-associated pUL34 to form the NEC, (F) further sequential interactions between several pUL31 and pUL34 molecules induce membrane wrapping until primary capsid envelopment is complete.

In summary, our data not only provide insights into the molecular function of HSV-1 pUL31 during translocation of nucleocapsids from the nucleoplasm to sites of primary envelopment. Given the conservation of the many players involved, the sequence of events described here seems to be applicable to all herpesviruses. Transit of nucleocapsids from the nucleoplasm to the INM is undoubtedly a highly complex process [2–8]. Numerous cellular and viral factors are expected to assist and accompany the nucleocapsids decorated with pUL31 in order to pave their way from the nuclear interior to the INM [33,72]. Thus, the results as well as the tools presented are invaluable to identify proteins and to decipher their function involved in nuclear translocation of capsids, a step decisive for propagation of herpesviruses and potentially other viruses relying on nuclear morphogenesis.

## Supporting Information

S1 Fig Physical interaction of pUL31 with importins and pUL34. (A) Interaction of pUL31 and pUL31-mp1mp2 with α importins KPNA2, KPNA4 and KPNA5 was tested by LUMIER assay. (B) Interaction of pUL31, pUL31-mp1mp2 and pSV40NLS-UL31-mp1mp2 with pUL34 lacking its C-terminal transmembrane domain (pUL34ΔTM) was tested by LUMIER assay.
(TIF)

S2 Fig UL32 is unaffected by UL31 mutagenesis. The coding regions of UL31 and UL32 overlap (Fig 4). To analyze the integrity of the UL32 locus during UL31 mutagenesis, Vero cells were transfected with BAC DNA of pHSV1(17^+^)Lox, LoxΔUL31 or Lox-UL31-mp1mp2 and analyzed at 20 hpt by IF using monoclonal antibodies recognizing ICP8, a marker for replication compartments, in combination with anti-pUL32 antibodies followed by secondary antibodies. Nuclei were visualized by DAPI. Analysis was performed by confocal microscopy. The scale bar corresponds to 10 μm.
(TIF)

S3 Fig In the course of infection, targeting of pUL31 to the nucleus is independent of an NLS. (A, B) To follow pUL31 and pUL34 in cells transfected with pHSV1(17^+^)Lox, LoxΔUL31, Lox-UL31-mp1mp2, Lox-UL31-mp1mp2 *rev*, Lox-SV40NLS-UL31-mp1mp2, or Lox-SV40NLS-UL31-mp1mp2 *rev*, Vero cells were transfected for 20 h and analyzed by IF using antibodies directed against VP5 hexons (mAb 8F5) in combination with anti-pUL31 antibodies (A) or anti-pUL34 antibodies (B) followed by Alexa 594- (A) or Alexa 555- (B), and Alexa 488-conjugated secondary antibodies (A). Nuclei were visualized by DAPI, confocal microscopy was applied for analysis. Each scale bar corresponds to 10 μm.
(TIF)

S4 Fig pUL31 lacking the N-terminal domain is partially retained in the cytoplasm but targets to nucleoplasmic sites of capsid assembly. (A) To determine the localization of pUL31 encoded by pHSV1(17^+^)Lox, Lox-UL31ΔN, or Lox-SV40NLS-UL31ΔN during infection, Vero cells were transfected with BAC DNA and analyzed at 20 hpt by IF using antibodies directed against VP5 hexons (mAb 8F5) together with anti-pUL31 antibodies followed by Alexa 555- (pUL31) and Alexa 488- (VP5) conjugated secondary antibodies. (B) To analyze the subcellular localization of the NEC component pUL34 in cells transfected with pHSV1(17^+^)Lox, Lox-UL31ΔN, or Lox-SV40NLS-UL31ΔN, Vero cells were transfected for 20 h and analyzed by IF using anti-pUL34 antibodies together with Alexa 488-conjugated secondary antibodies. Nuclei were visualized by DAPI, confocal microscopy was applied for analysis. Each scale bar corresponds to 10 μm.
(TIF)

S5 Fig Subcellular distribution of pUL31 and nucleocapsids in cells infected with HSV1(17^+^)Lox-UL31-hbpmp1mp2. To determine the localization of pUL31-hbpmp1mp2 in detail, Vero cells were infected with HSV1(17^+^)Lox or Lox-UL31-hbpmp1mp2 using an MOI of 1 and analyzed at 12 hpi by IF using anti-pUL31 antibodies in combination with antibodies directed against VP5 hexons (mAb 8F5) followed by Alexa 555- and Alexa 488-conjugated secondary antibodies, respectively. For analysis, confocal microscopy was applied. The scale bar corresponds to 10 μm.
(TIF)

S6 Fig Subcellular distribution of pUL34 and nucleocapsids in cells infected with HSV1(17^+^)Lox-UL31-hbpmp1mp2. To compare the localization of pUL34 during infection with HSV1(17^+^)Lox or Lox-UL31-hbpmp1mp2, cells were infected using an MOI of 1 and analyzed at 12 hpi by IF using anti-pUL34 antibodies in combination with antibodies directed against VP5 hexons (mAb 8F5) followed by Alexa 555- and Alexa 488-conjugated secondary antibodies, respectively. For analysis, confocal microscopy was applied. The scale bar corresponds to 10 μm.
(TIF)
