# Supplementary figures and images for: The Herpes Simplex Virus Protein pUL31 Escorts Nucleocapsids to Sites of Nuclear Egress, a Process Coordinated by Its N-Terminal Domain
Source: PLoS Pathog. 2015 Jun 17;11(6):e1004957. doi: 10.1371/journal.ppat.1004957 (PMC4471197; doi:10.1371/journal.ppat.1004957)

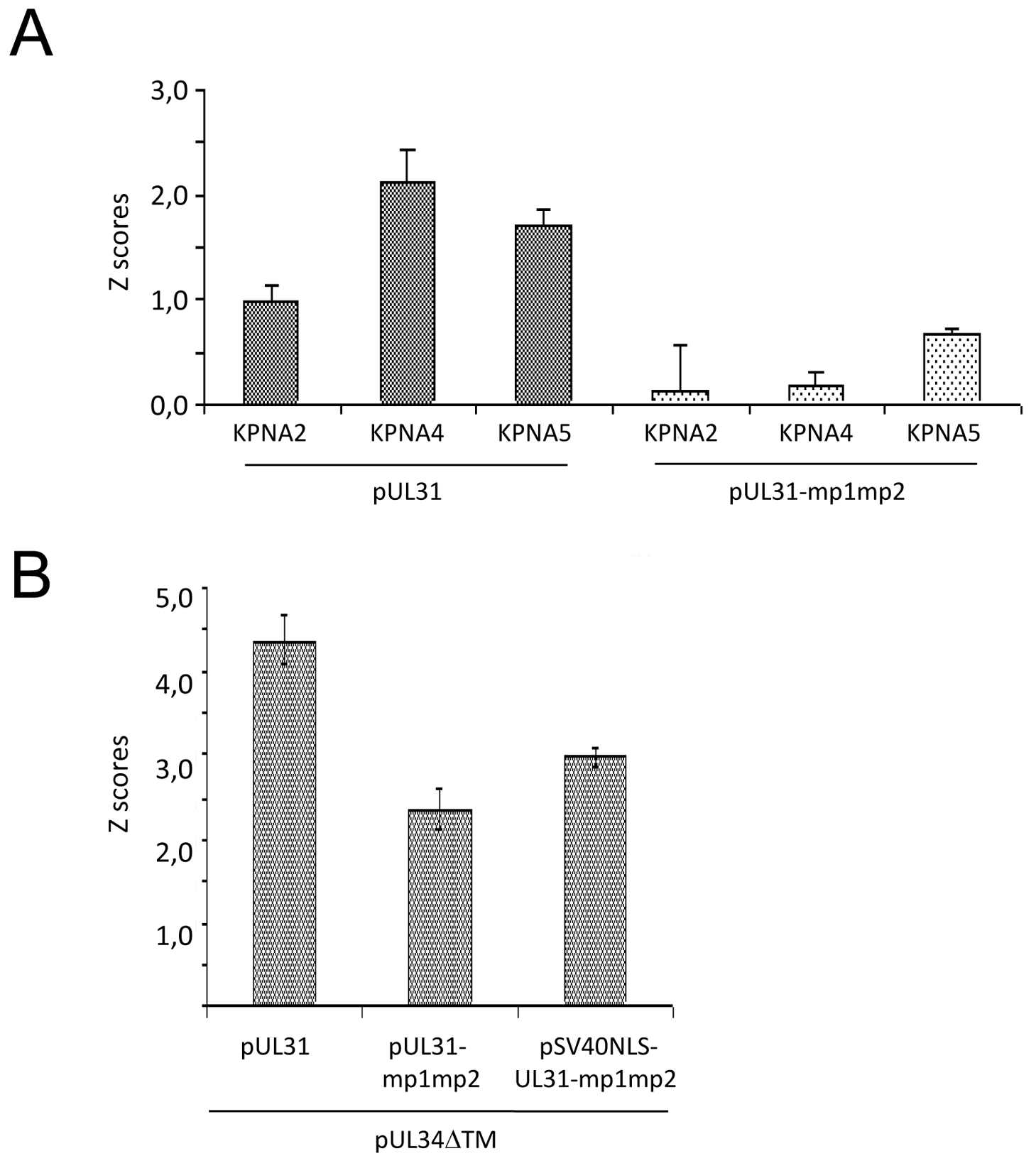

Supplement: S1 Fig — (A) Interaction of pUL31 and pUL31-mp1mp2 with α importins KPNA2, KPNA4 and KPNA5 was tested by LUMIER assay. (B) Interaction of pUL31, pUL31-mp1mp2 and pSV40NLS-UL31-mp1mp2 with pUL34 lacking its C-terminal transmembrane domain (pUL34ΔTM) was tested by LUMIER assay. (TIF) [file ppat.1004957.s001.tif]

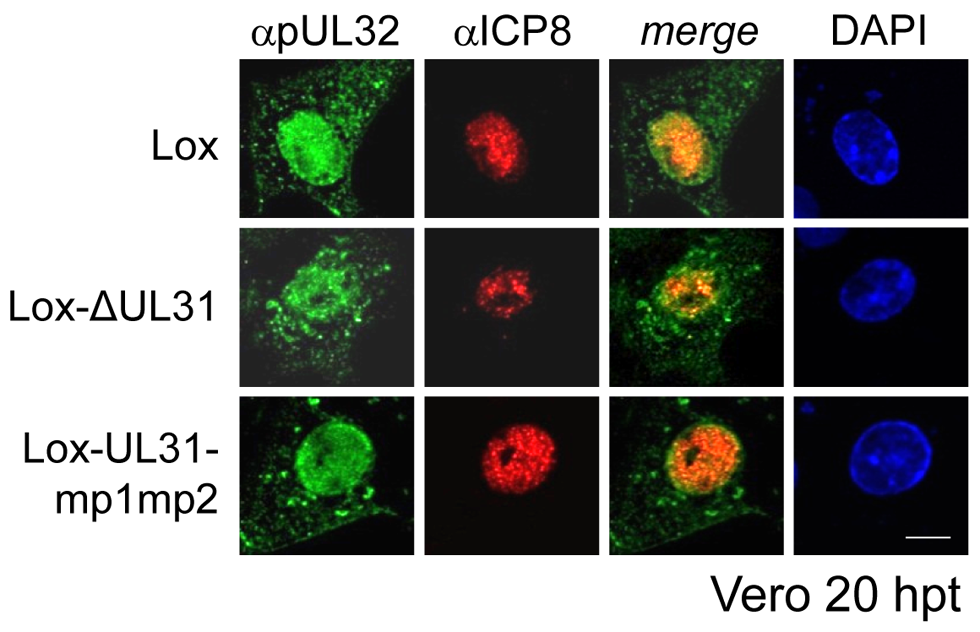

Supplement: S2 Fig — The coding regions of UL31 and UL32 overlap (Fig 4). To analyze the integrity of the UL32 locus during UL31 mutagenesis, Vero cells were transfected with BAC DNA of pHSV1(17+)Lox, LoxΔUL31 or Lox-UL31-mp1mp2 and analyzed at 20 hpt by IF using monoclonal antibodies recognizing ICP8, a marker for replication compartments, in combination with anti-pUL32 antibodies followed by secondary antibodies. Nuclei were visualized by DAPI. Analysis was performed by confocal microscopy. The scale bar corresponds to 10 μm. (TIF) [file ppat.1004957.s002.tif]

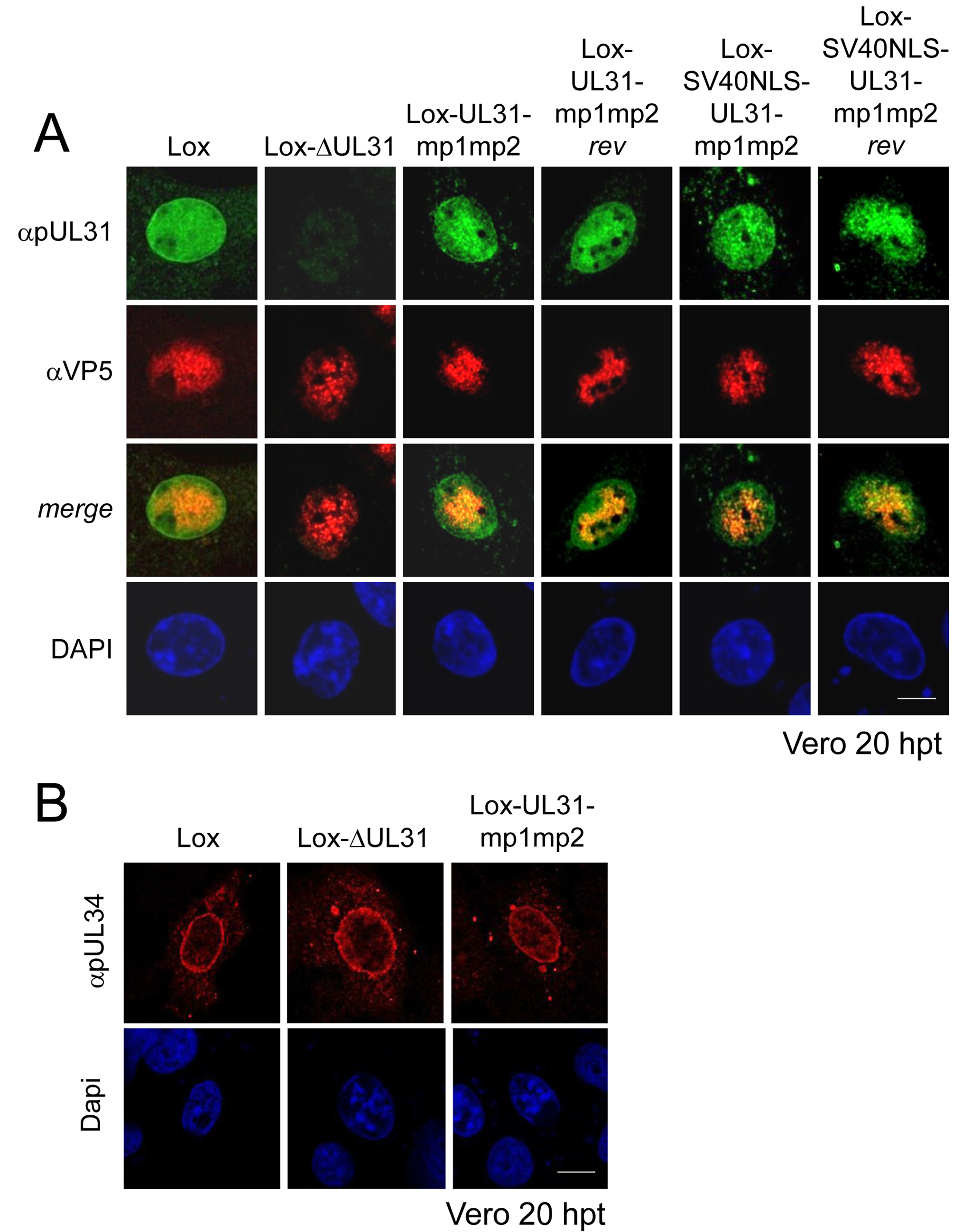

Supplement: S3 Fig — (A, B) To follow pUL31 and pUL34 in cells transfected with pHSV1(17+)Lox, LoxΔUL31, Lox-UL31-mp1mp2, Lox-UL31-mp1mp2 rev, Lox-SV40NLS-UL31-mp1mp2, or Lox-SV40NLS-UL31-mp1mp2 rev, Vero cells were transfected for 20 h and analyzed by IF using antibodies directed against VP5 hexons (mAb 8F5) in combination with anti-pUL31 antibodies (A) or anti-pUL34 antibodies (B) followed by Alexa 594- (A) or Alexa 555- (B), and Alexa 488-conjugated secondary antibodies (A). Nuclei were visualized by DAPI, confocal microscopy was applied for analysis. Each scale bar corresponds to 10 μm. (TIF) [file ppat.1004957.s003.tif]

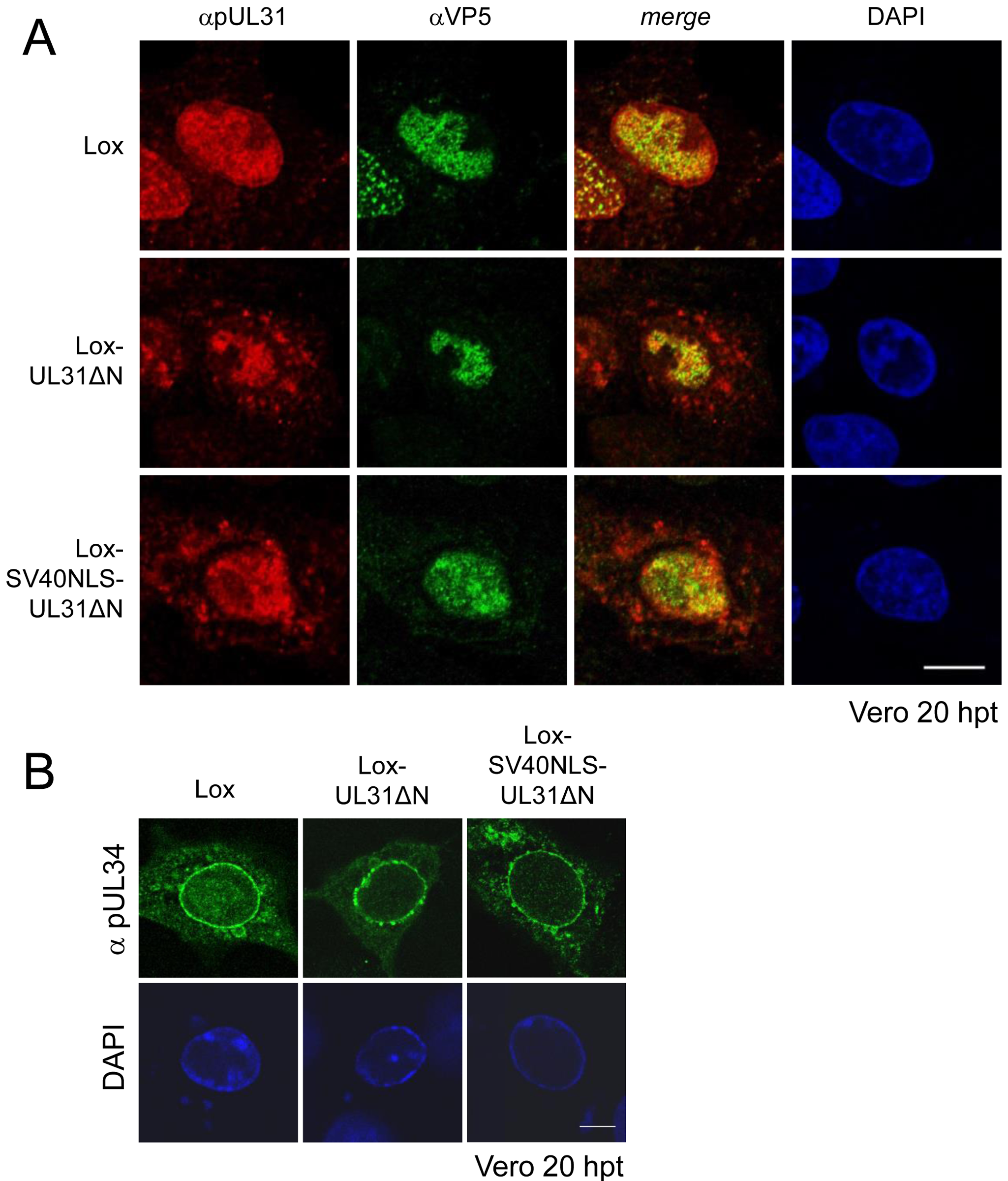

Supplement: S4 Fig — (A) To determine the localization of pUL31 encoded by pHSV1(17+)Lox, Lox-UL31ΔN, or Lox-SV40NLS-UL31ΔN during infection, Vero cells were transfected with BAC DNA and analyzed at 20 hpt by IF using antibodies directed against VP5 hexons (mAb 8F5) together with anti-pUL31 antibodies followed by Alexa 555- (pUL31) and Alexa 488- (VP5) conjugated secondary antibodies. (B) To analyze the subcellular localization of the NEC component pUL34 in cells transfected with pHSV1(17+)Lox, Lox-UL31ΔN, or Lox-SV40NLS-UL31ΔN, Vero cells were transfected for 20 h and analyzed by IF using anti-pUL34 antibodies together with Alexa 488-conjugated secondary antibodies. Nuclei were visualized by DAPI, confocal microscopy was applied for analysis. Each scale bar corresponds to 10 μm. (TIF) [file ppat.1004957.s004.tif]

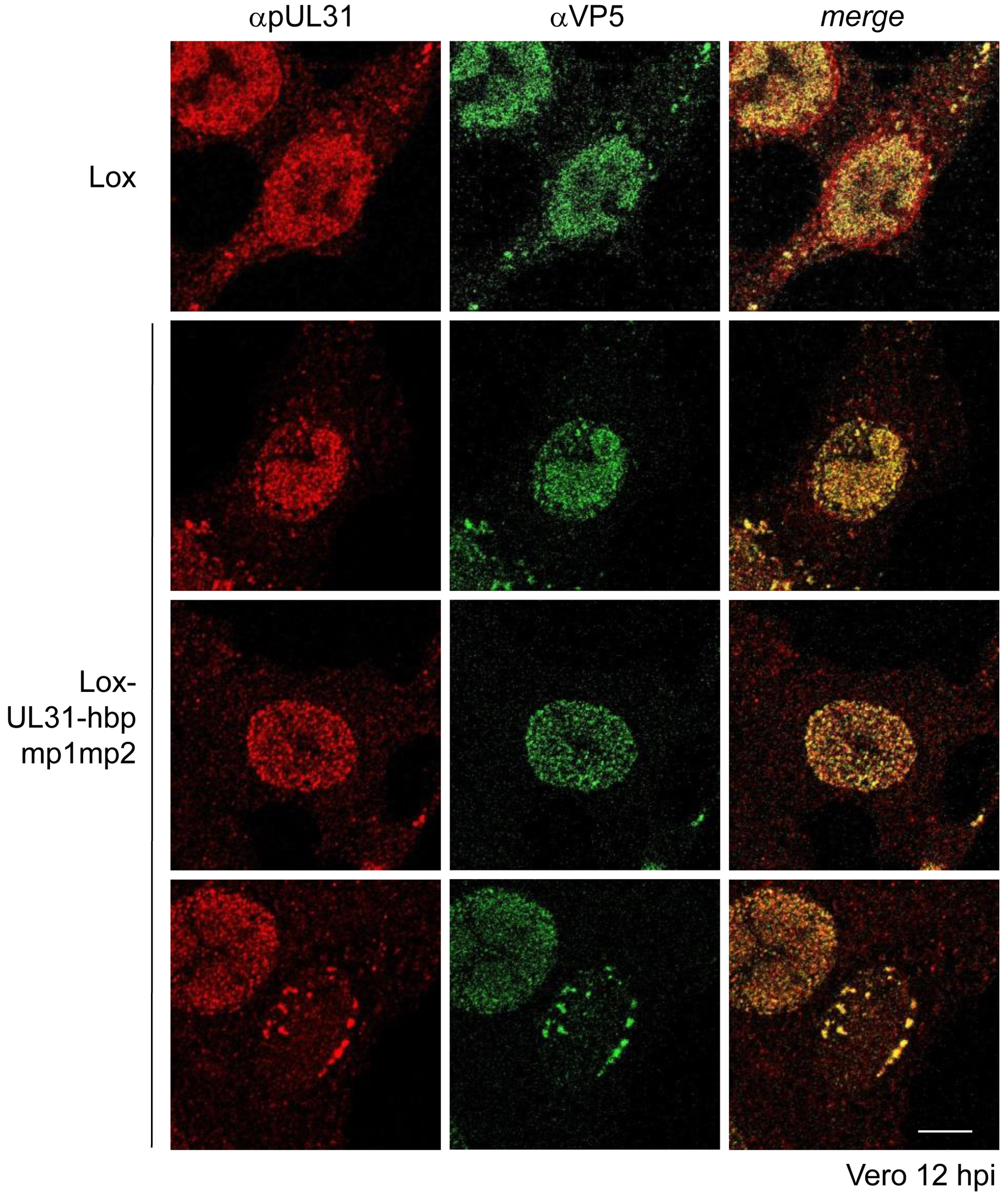

Supplement: S5 Fig — To determine the localization of pUL31-hbpmp1mp2 in detail, Vero cells were infected with HSV1(17+)Lox or Lox-UL31-hbpmp1mp2 using an MOI of 1 and analyzed at 12 hpi by IF using anti-pUL31 antibodies in combination with antibodies directed against VP5 hexons (mAb 8F5) followed by Alexa 555- and Alexa 488-conjugated secondary antibodies, respectively. For analysis, confocal microscopy was applied. The scale bar corresponds to 10 μm. (TIF) [file ppat.1004957.s005.tif]

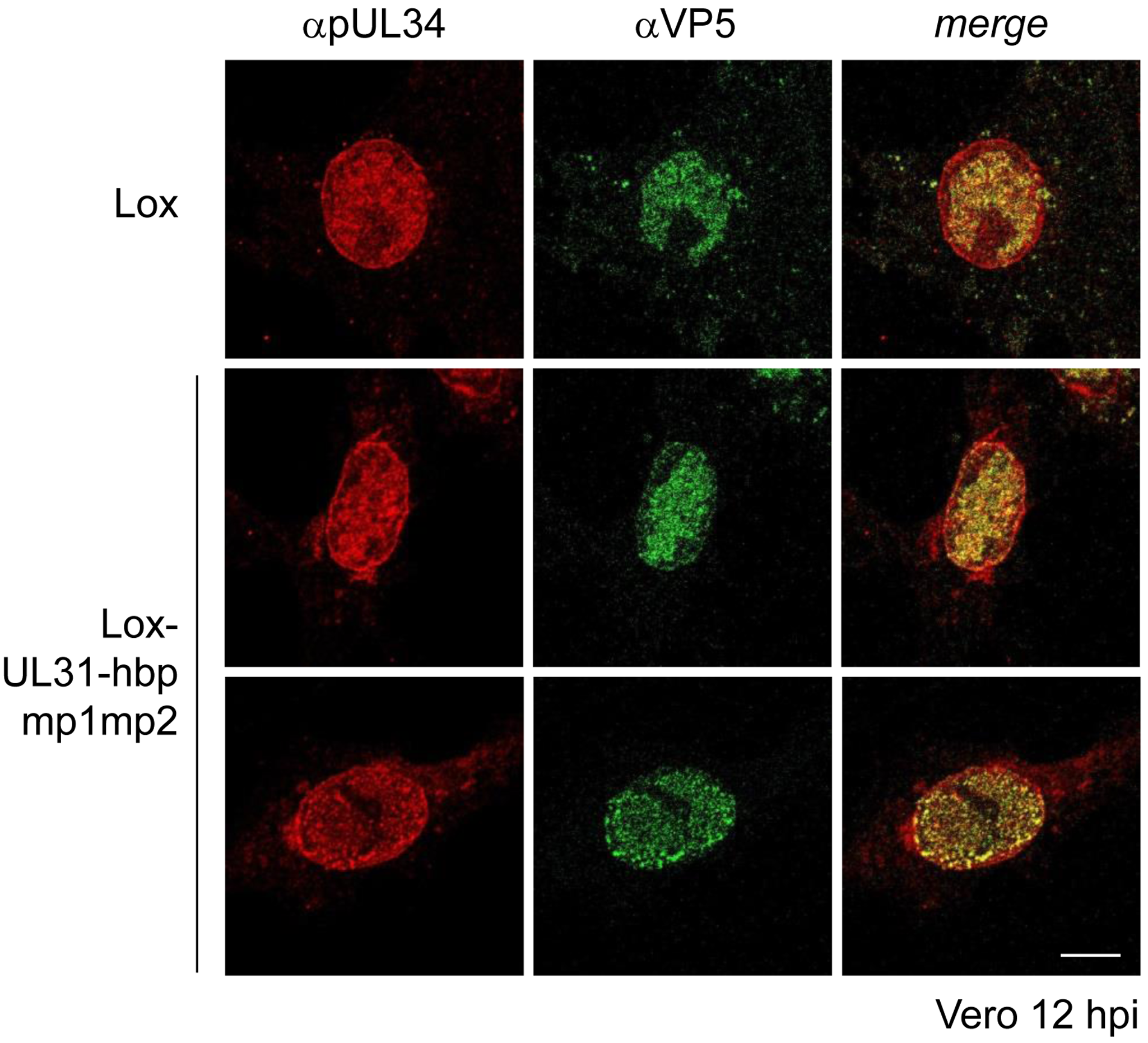

Supplement: S6 Fig — To compare the localization of pUL34 during infection with HSV1(17+)Lox or Lox-UL31-hbpmp1mp2, cells were infected using an MOI of 1 and analyzed at 12 hpi by IF using anti-pUL34 antibodies in combination with antibodies directed against VP5 hexons (mAb 8F5) followed by Alexa 555- and Alexa 488-conjugated secondary antibodies, respectively. For analysis, confocal microscopy was applied. The scale bar corresponds to 10 μm. (TIF) [file ppat.1004957.s006.tif]
